# A potential biomarker for clinical atherosclerosis: A novel insight derived from myosin heavy chain 10 promoting transformation of vascular smooth muscle cells

**DOI:** 10.1002/ctm2.672

**Published:** 2022-01-24

**Authors:** Ling‐bing Meng, Jian‐yi Li, Hong‐xuan Xu, Di‐shan Wu, Meng‐jie Shan, Yu‐hui Chen, Jia‐pei Xu, Long‐teng Liu, Zuoguan Chen, Yong‐jun Li, Tao Gong, De‐ping Liu

**Affiliations:** ^1^ Department of Cardiology Beijing Hospital National Center of Gerontology Chinese Academy of Medical Sciences Institute of Geriatric Medicine Beijing China; ^2^ Graduate School Chinese Academy of Medical Sciences & Peking Union Medical College Beijing China; ^3^ Department of Plastic Surgery Peking Union Medical College Hospital Beijing China; ^4^ Department of Neurology Beijing Hospital National Center of Gerontology Institute of Geriatric Medicine Chinese Academy of Medical Sciences Beijing China; ^5^ Department of Pathology Beijing Hospital National Center of Gerontology Institute of Geriatric Medicine Chinese Academy of Medical Sciences Beijing China; ^6^ Department of Vascular Surgery Beijing Hospital National Center of Gerontology Institute of Geriatric Medicine Chinese Academy of Medical Sciences Beijing China

Dear Editor,

The expression of myosin heavy chain 10 (MYH10) in atherosclerosis (AS) was positively correlated with the number of vascular smooth muscle cells (VSMCs), which were negatively related to the AS measured by intima‐media thickness (IMT). MYH10 might be a potential biomarker for clinical AS, which was the formation of fibrous fatty lesions in the arterial wall. Over time, atherosclerotic plaques become more fibrotic.^1^ Advanced atherosclerotic plaques can invade arterial cavities, block blood flow, or form clots, resulting in tissue ischemia.^2^ Although some progress has been made in the treatment of AS, drugs and arterial interventional therapy cannot target the pathogenesis and progression of the disease, and there are risks of postoperative thrombosis and restenosis.^3^, ^4^ Because of the complexity of its pathogenesis, gene therapy is still in the stage of basic research and is still a serious challenge in modern medicine.

VSMCs are one of the main cells that form the tissue structure of the vascular wall and maintain vascular tension. Studies have shown that the origin, phenotypic transformation, apoptosis, and calcification of VSMCs are closely related to AS.^5^ Some scholars believe that phenotypic transformation of VSMCs plays an important role in the formation of AS, and inhibition of phenotypic transformation of VSMCs has a protective effect on arteries.^6^ Under normal conditions, VSMCs in the arterial middle layer can express smooth muscle cell markers, such as myosin heavy chain 11 (MYH11), MYH9, MYH10, and α‐smooth muscle actin (α‐SMA), but the ability of VSMCs in the arterial middle layer with AS to express these markers is decreased.^7^

MYH10 (Myosin Heavy Chain 10) is a member of the Myosin superfamily.^8^ This protein represents a conventional non‐muscle myosin. Myosin is an actin‐dependent motor protein with a variety of functions, including regulation of cell division, cell movement, and cell polarity.^9^ However, the role of MYH10 and α‐SMA in the development of AS remains unclear.

Therefore, this study aims to explore the potential role of MYH10 in AS by weighted gene co‐expression network analysis, gene set enrichment analysis, and analysis of AS‐infiltrating immune cells. Furthermore, the mechanism of the interaction between MYH10 and α‐SMA on AS was analysed through animal models of AS, and clinical specimen experiments.

The hematoxylin‐eosin staining (20×, 100×, 200×) results showed that the intima of the arterial sections in the normal group was smooth and muscle fibres were neatly arranged. However, for the clinical AS sample, muscle fibres were arranged irregularly, and the organizational structure is of evacuation (Figure 1A). The immunohistochemical assay (100×, 200×, 400×) results showed that the MYH10 was clearly expressed in the media of the arteries, and there were few expressions in the intima membrane in the normal group (The brown‐yellow colour represents the expression of the MYH10 molecule, and the blue colour represents the nucleus). However, in the clinical AS samples, the MYH10 was down‐expressed significantly in the media of arteries (Figure 1B). Furthermore, the immunofluorescence assay also manifested that compared with the normal group, the expression of MYH10 in the media of the arteries was lower in the clinical AS group, and there were very few expressions in the intima membrane (the red colour represents the expression of the MYH10 molecule, and the blue colour represents the nucleus; Figure 1C). Quantitative analysis of expression of MYH10 IHC‐Score manifested that the expression of MYH10 was significantly lower in the clinical AS tissues than in the control group (Figure 1D). The RT‐PCR results indicated that the relative expression level of MYH10 mRNA was significantly decreased in clinical AS artery compared with control artery tissue samples without AS (*p* < .05, Figure 1E). Western blotting analysis showed that the expression of MYH10 proteins was down‐regulated in the clinical AS samples compared with the control group (Figure 1F).

**FIGURE 1:**
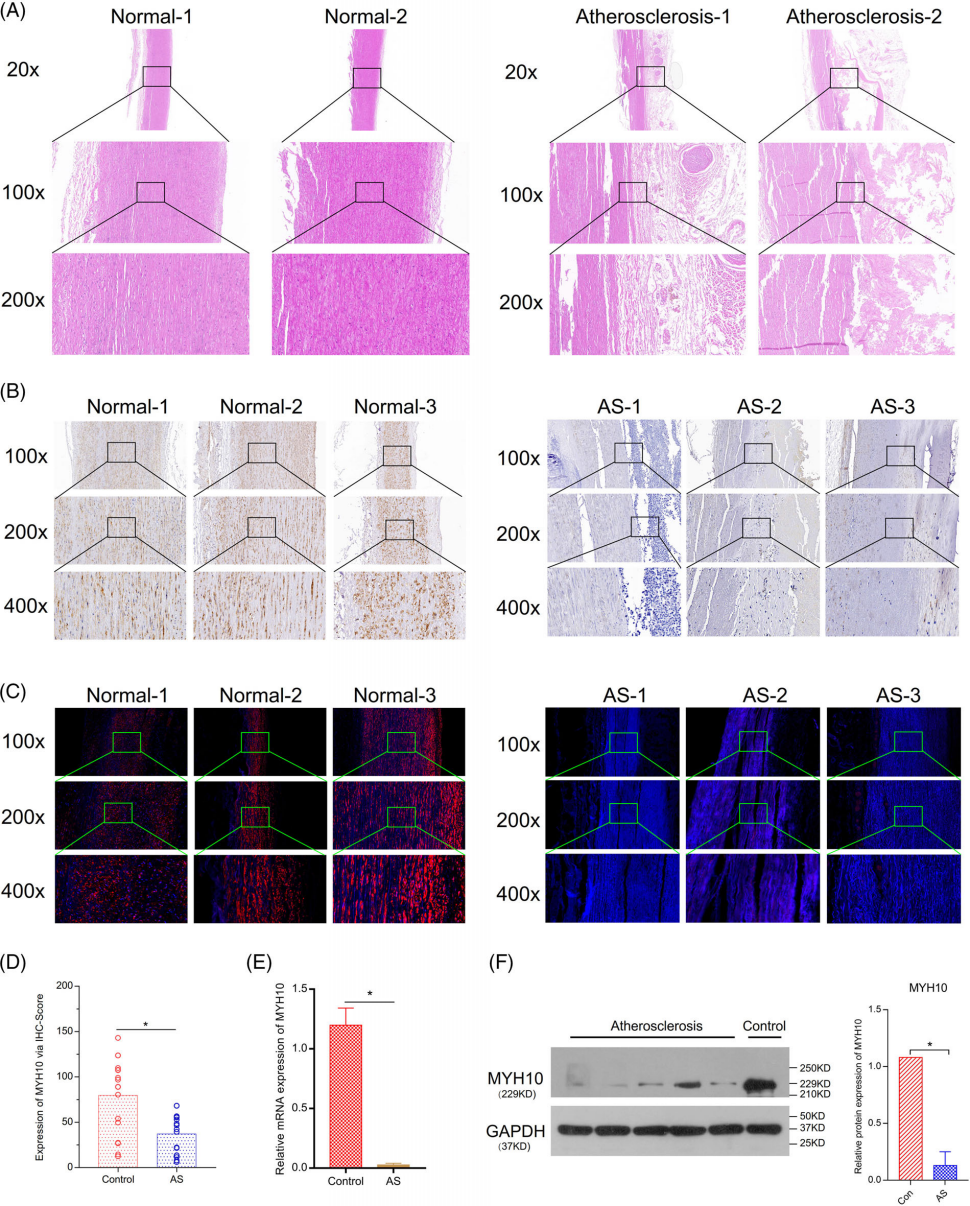
Verification for the role of myosin heavy chain 10 (MYH10) based on clinical atherosclerosis (AS) samples. (A) The hematoxylin‐eosin (HE) staining (20×, 100×, 200×) manifesting the histopathology of normal artery and clinical atherosclerosis. (B) The immunohistochemical assay (100×, 200×, 400×) showed that the MYH10 was down‐expressed significantly in the media of arteries in the clinical AS samples compared with the normal group (the brown‐yellow colour represents the expression of the MYH10 molecule, and the blue colour represents the nucleus). (C) The immunofluorescence assay showed that the MYH10 was down‐expressed significantly in the media of arteries in the clinical AS samples compared with the normal group (the red colour represents the expression of the MYH10 molecule, and the blue colour represents the nucleus). (D) Quantitative analysis of expression of MYH10 IHC‐Score. (E) The RT‐PCR results indicated the relative expression level of MYH10 mRNA. (F) Western blotting analysis showed that the expression of MYH10 proteins was downregulated in the clinical AS samples compared with the control group

The immunohistochemistry and immunofluorescence assays (100×, 200×, 400×) showed that the expression of α‐SMA protein in the clinical AS group was lower than that in the normal group. In immunohistochemistry, the brown‐yellow colour represents the expression of the α‐SMA molecule, and the blue colour represents the nucleus. The red colour represents the expression of the α‐SMA molecule, and the blue colour represents the nucleus (Figure 2A,B).

**FIGURE 2:**
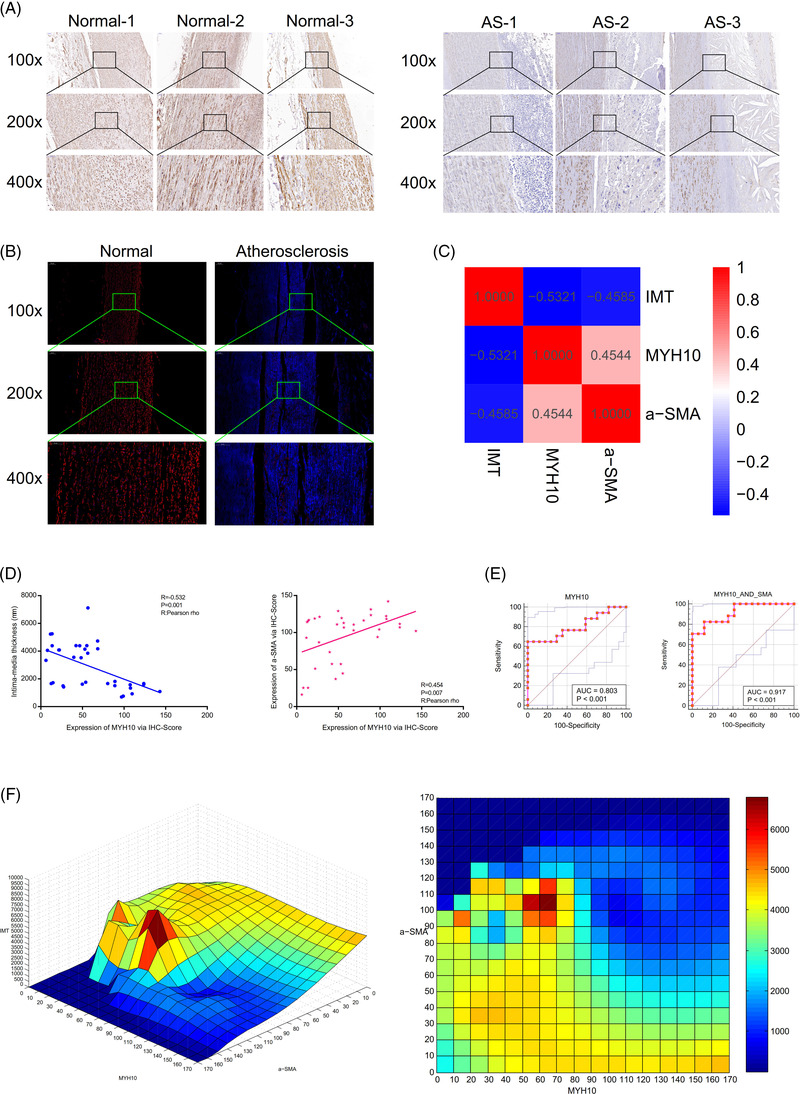
The expression level of α‐smooth muscle actin (α‐SMA), and correlation analysis of the intima‐media thickness (IMT), α‐SMA, and myosin heavy chain 10 (MYH10), and cubic spline interpolation algorithm in the clinical atherosclerosis group. (A) The immunohistochemistry (100×, 200×, 400×) showed that the expression of α‐SMA protein in the clinical atherosclerosis group was lower than that in the normal group. (B) Immunofluorescence assay manifesting the expression of α‐SMA protein. (C,D) There were strong correlations among the IMT, MYH10 and α‐SMA. (E) The expression of MYH10 could predict the IMT sensitively and specifically (area under the curve [AUC] = .803, *p* < .001). Receiver operator characteristic (ROC) curves were constructed to determine the joint effect of MYH10 and α‐SMA on the IMT, with the degree of confidence judged by the AUC = .917 (*p* < .001). (F) Cubic spline interpolation algorithm showed that MYH10 and α‐SMA might be the high‐risk warning indicator of IMT of the atherosclerosis (AS)

Through the Pearson rho analysis, there existed a strong negative relationship between the IMT and MYH10 (*p* = .001, *R* = ‐.532). The IMT was also negatively related to α‐SMA (*p* < .001, *R* = −.4585). In addition, a strong relationship existed between α‐SMA and MYH10 (*p* = .007, *R* = .454). A heatmap showed that there were strong correlations among the IMT, MYH10, and α‐SMA (Figure 2C,D). The expression of MYH10 could predict the IMT sensitively and specifically (area under the curve [AUC] = .803, *p* < .001). Receiver operator characteristic curves were constructed to determine the effect of MYH10 and α‐SMA on the IMT, with the degree of confidence judged by the AUC = .917 (*p* < .001, Figure 2E).

Results of the cubic spline interpolation algorithm showed that MYH10 and α‐SMA might be the high‐risk warning indicator of IMT of the AS (Figure 2F). After training by neural network model, the best training performance is 0.036573 at epoch 3000 (Figure 3A). The relativity of the model (built by MYH10, α‐SMA and IMT) is 0.92506 (Figure 3B). Furthermore, the model could be verified well, and there exists a tiny error between raw data and forecast data (Figure 3C,D). The above result could demonstrate that the joint effect of MYH10 and α‐SMA would be predictive parameters of IMT in the clinical AS.

**FIGURE 3:**
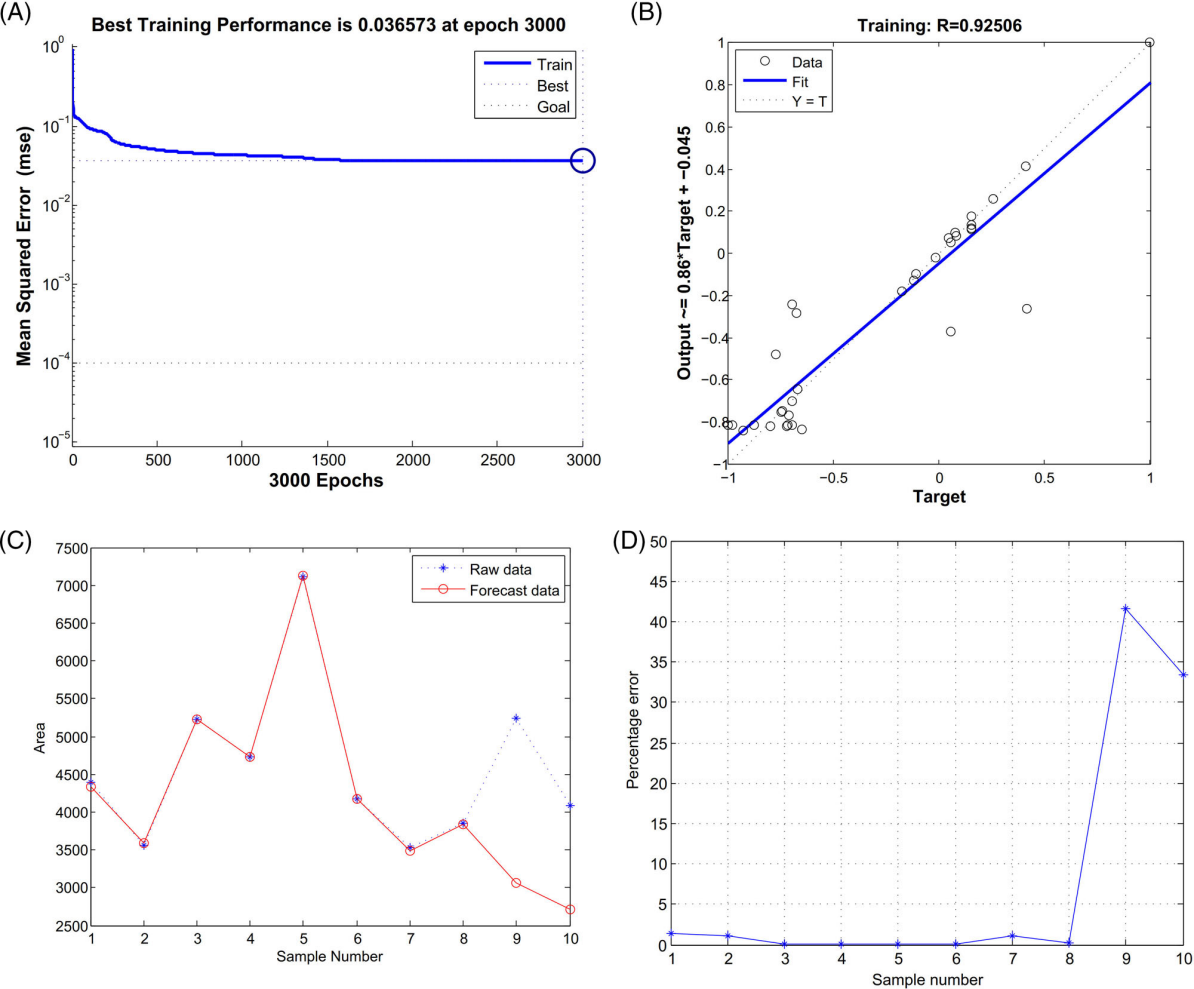
Prediction of intima‐media thickness (IMT) of the clinical atherosclerosis (AS) through neural network model. (A) The best training performance is 0.036573 at epoch 3000. (B) The relativity of the model (built by MYH10, α‐smooth muscle actin [α‐SMA] and IMT) is 0.92506. (C,D) The model could be verified well, and there exists a tiny error between raw data and forecast data

Through the molecular docking study, the lowest binding energy of optimal complex conformation was −7.29 kcal/mol. The binding mode between the MYH10 and α‐SMA was drawn (Figure 4A). Through the co‐immunoprecipitation assay, the MYH10 and α‐SMA were co‐expressed in the artery (Figure 4B,C).

**FIGURE 4:**
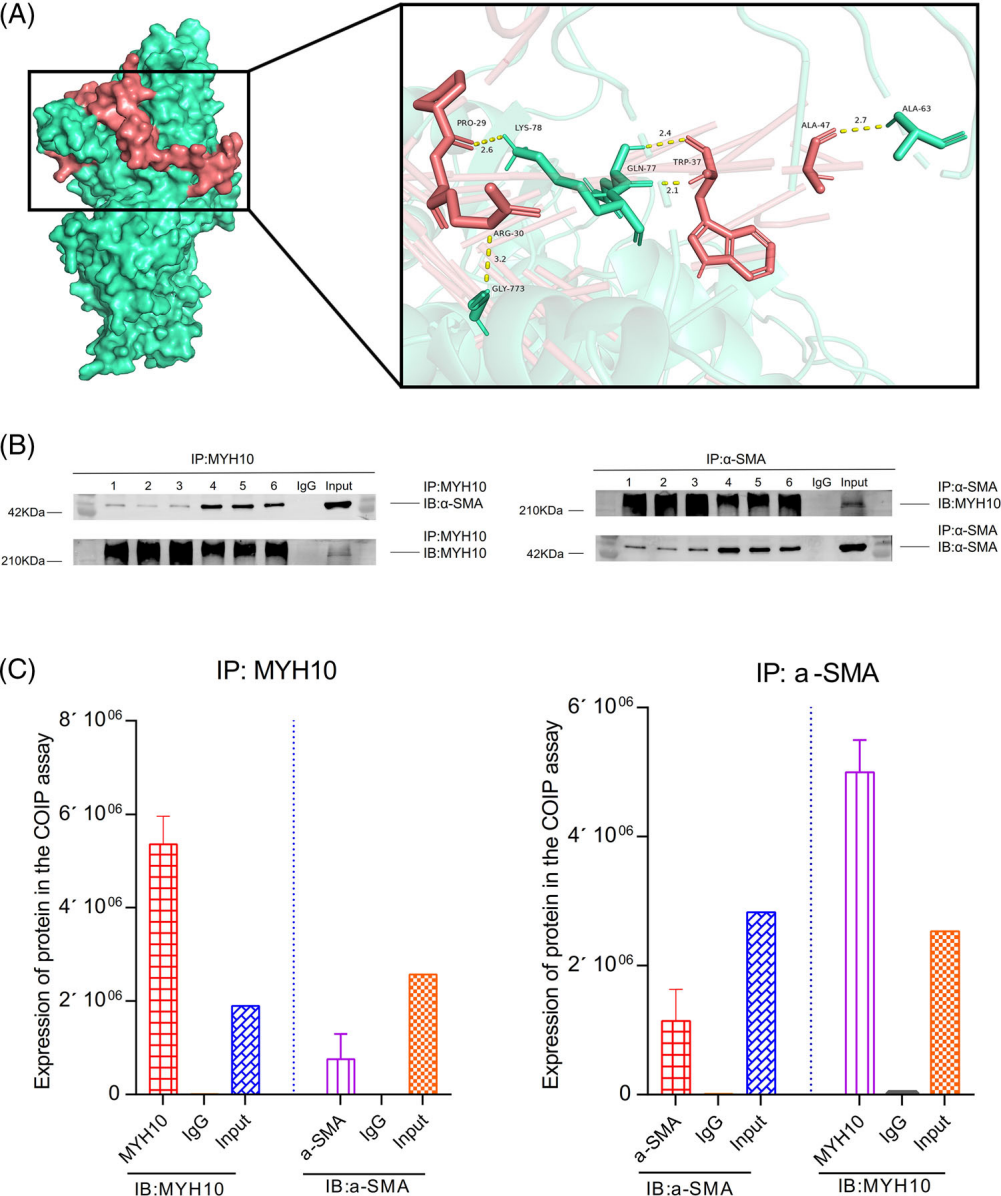
Co‐expression analysis between myosin heavy chain 10 (MYH10) and α‐smooth muscle actin (α‐SMA). (A) Molecular docking study of MYH10 and α‐SMA. (B，C) The co‐immunoprecipitation (Co‐IP) experiment manifested that the MYH10 and α‐SMA were co‐expressed in the artery

In summary, the expression of MYH10 was positively correlated with the number of VSMCs (measured by α‐SMA), which were negatively related to the AS measured by IMT. When MYH10 expression was low, the proliferation of VSMCs was decreased. Moreover, changes of VSMCs might promote the intima thickening and the formation of AS.

## CONSENT FOR PUBLICATION

None.

## CONFLICT OF INTEREST

The authors declare that they have no conflict of interest.

## Supporting information

Supporting information
