# Supplementary material for: A potential biomarker for clinical atherosclerosis: A novel insight derived from myosin heavy chain 10 promoting transformation of vascular smooth muscle cells
Source: Clin Transl Med. 2022 Jan 24;12(1):e672. doi: 10.1002/ctm2.672 (PMC8787098; doi:10.1002/ctm2.672)
Supplement: Supplementary file 1 — Supporting information [file CTM2-12-e672-s001.docx]

**Supplemental materials:**

**A potential biomarker for clinical** **atherosclerosis: a novel insight derived from myosin heavy chain 10 promoting transformation of vascular smooth muscle cells**

**1.Introduction:**

At present, atherosclerotic cardiovascular disease is still the major cause of vascular disease worldwide, causing acute coronary syndrom, including myocardial infarction and stable angina pectoris, as well as ischemic cerebrovascular disease and aneurysm formation. Heart disease, the most common cause of which is atherosclerotic disease in the coronary arteries, and stroke are the two leading causes of death worldwide^1,2^. In 2015, more than 17 million people died of cardiovascular disease, accounting for 31 percent of all deaths worldwide^3^. When atherosclerosis affects the peripheral arteries, it can lead to intermittent claudication, ulcers, and gangrene, affecting the patient's quality of life. The occurrence and development of AS is a long-term and complex pathological process. With the continuous development of science and technology, it is still necessary to explore new treatment methods from the perspective of molecular mechanism to fundamentally solve the formation and development of AS, so AS to prevent and cure atherosclerotic cardiovascular and cerebrovascular diseases.

**2. Materials and methods**

**2.1 Bioinformatic analysis of the role of MYH10 on AS**

2.1.1 The datasets of AS

In this study, the proﬁles of AS GSE43292 was downloaded from the GEO database (<http://www.ncbi.nlm.nih.gov/geo/>), which were generated using GPL6244. Additionally, they were paired, including 32 AS samples and 32 normal samples, which were used to identify differently expressed genes (DEGs) between AS and normal tissues.

2.1.2 Scoring the microenvironment

ESTIMATE is mainly based on single sample Gene Set Enrichment Analysis, scoring two gene sets of stromal and immune. In this study, we used the ESTIMATE algorithm to score the microenvironment.

2.1.3 Statistical Analysis

The analyses in this study were conducted using MedCalc software. Associations between micro score and the status of sample was tested by ROC curve.

2.1.4 Screening of DEGs

R packages “limma” was employed to perform probe summarization and background correction of GSE43292. Use the stromal score, immune score and disease as grouping basis for differential gene screening. The Benjamini-Hochberg method was used to adjust the original p-values. Fold-changes (FC) were calculated using the false discovery rate (FDR). The cut-off criteria for DEGs were FDR < 0.05.

2.1.5 Weighted gene co-expression network analysis (WGCNA)

The WGCNA analysis of all genes in GSE43292 was performed using R package “WGCNA”. A co-expression network for all genes was constructed, and 32 AS samples and 32 normal samples were used for WGCNA analysis. The adjacency matrix was created by samples, and then it was transformed into a topological overlap matrix (TOM). Genes were divided into different gene modules using TOM-based difference measurement. The minimal gene module > 200 and the threshold to merge similar modules=0.1 were used to search modules that play an important role in AS. At the same time, WGCNA also predicted the interconnection of genes in the module, and then we imported the data into cytoscape software to search for predicted key genes. Key genes are derived through four algorithms (Closeness, MCC, MNC and Radiality) and drawn by Funrich software. Cytoscape software can provide biological network analysis and two-dimensional (2D) visualization for biologists. Funrich software is a comprehensive biological analysis tool.

2.1.6 Construction and analysis of protein-protein interaction (PPI) network

STRING database (http://string‑db.org/) aims to collect, score and integrate all publicly available sources of protein-protein interaction information, and supplement these sources through computational predictions. In this study, PPI networks of predicted key genes were constructed by STRING database (confidence score > 0.4). Subsequently, PPI networks was visualized and analyzed by Cytoscape software. Four algorithms (Closeness, MCC, MNC and Radiality) were applied to search hub genes

2.1.7 Functional enrichment analysis

The Gene Ontology (GO) and Kyoto Encyclopedia of Genes and Genomes (KEGG) analyses are computational methods that evaluate gene functions and biological pathways. Metascape (<http://metascape.org/gp/index.html>) database can provide a comprehensive gene list annotation and analysis resource. In this study, GO and KEGG analyses of blue module were performed to explore the potential mechanism in AS.

2.1.8 Single gene analysis

Single gene difference analysis and paired difference analysis are performed by R.

2.1.9 GSEA analysis

Gene Set Enrichment Analysis (GSEA) is a computational method that could execute GO and KEGG analysis with complete genome. In our study, We grouped the samples according to the level of expression of MYH10, and the GO and KEGG analysis of the complete genome were performed by GSEA.

2.1.10 Evaluation of AS-inﬁltrating immune cells

CIBERSORT is a computational approach developed by Chen et al, which could characterize tumor-inﬁltrating immune cells in tumor samples profiled by microarray or RNA-Seq and accurately estimate the immune composition of a tumor biopsy. The gene expression signature matrix of 547 marker genes that could quantify and define 22 immune cell subtypes were download from the CIBERSORT web portal (<http://cibersort.stanford.edu/>).

The 22 immune cells included B cells naïve, B cells memory, Plasma cells, T cells CD8, T cells CD4 naive, T cells CD4 memory resting, T cells CD4 memory activated, T cells follicular helper, T cells regulatory (Tregs), T cells gamma delta, NK cells resting, NK cells activated, Monocytes, Macrophages M0, Macrophages M1, Macrophages M2, Dendritic cells resting, Dendritic cells activated, Mast cells resting, Mast cells activated, Eosinophils and Neutrophils.

In our study, the distribution and number of immune cells between AS and normal were calculated via CIBERSORT (P<0.05).

2.1.11 Statistical Analysis of AS-inﬁltrating immune cells

The analyses in this study were constructed using Perl and R software (version 3.5.3). All case that P value < 0.05 with CIBERSORT analysis were included in the subsequent analysis. Wilcox test were employed to detect the differential infiltrations of the 22 immune cell types in the GSE43292. Correlations among different immune cells were tested by corrplot R package.

2.2 Verification via atherosclerosis animal model and clinical samples

2.2.1 Construction of the atherosclerosis animal model

Twenty New Zealand white rabbits (3-month-old) were randomly classified into two groups, including control group (CON group, n=10), and atherosclerotic group (AS group, n=10). The detailed information could be found in the previous publication^4^.

2.2.2 Clinical AS samples

2.2.2.1 Characteristics of patients

There were 34 samples obtained for this study, comprising 17 samples with atherosclerosis and 17 samples without atherosclerosis. The samples were from patients who received treatment in the Beijing Hospital between January 2018 and April 2021. All the patients underwent surgery in the vascular surgery department or the pathology department. There were 17 atherosclerosis tissue samples obtained from the atherosclerosis group and 17 normal arterial wall tissue samples collected from the control group. Basic information for all patients was carefully recorded.

2.2.2.2 Inclusion and exclusion criteria

The inclusion criteria for the experiment group were: aged 18 to 110 years; patients with atherosclerosis; patients who underwent surgery in the vascular surgery or the pathology department; consent had been obtained from the patient and their family.

The inclusion criteria for the control group were: aged 18 to 110 years; patients clinically diagnosed as needing amputation or an organ transplant, or patients who have had an arterial blood vessel transplant; individuals without atherosclerosis.

Exclusion criteria included: age < 18 or > 110 years; patients with poor wound healing or infection; patients with severely weakened cardiopulmonary function; and patients with high preoperative intracranial pressure, encephalocele, and intracranial infection.

2.2.3 The experimental assay

2.2.3.1 Hematoxylin-eosin (HE) staining

Removing the embedding agent and rehydration of undecalcified bone sections in ethylene glycol ethyl ether acetate (Macklin, E808814-2.5L) and alcohol. hematoxylin dyeing solution stains 8-10minutes. Then washing with running water, differentiated with differentiation solution and blue returning liquid to return blue, washing with running water every time. Respectively dehydrated sections with 85% and 95% ethanol absolute for 5mintues and stain 8-10min in eosin solution. Finally, ethanol absolute to dehydration, xylene to transparency, neutral balsam to mount sections. Then collect the images and analyze under optical microscopy (NIKON, Nikon Eclipse E100) that the nucleus was blue and the cytoplasm was red. The intima-media thickness (IMT) was measured using Case Viewer 2.4 software (3DHISTECH (Hungary) in HE stained sections of artery.

2.2.3.2 Immunohistochemical assay

The samples were fixed with 4% paraformaldehyde. Using citric acid (PH9.0) antigen retrieval buffer (Servicebio G1203, Wuhan, China) to antigen retrieval. Incubating the sections with 3% hydrogen peroxide (room temperature, darkness) for 25 minutes to block endogenous peroxidase. Then 3%BSA is used to seal for 30 minutes at room temperature. Removing the blocking solution, adding MYH10 antibody (dilution rate=1:1000, nbp1-55146, Novus, Colorado, USA) to the sections, incubating overnight at 4℃. Washing with PBS (PH7.4) (three times, 5min/time), the sections are slightly shaken and dried, adding the secondary antibody (HRP labeled) (dilution rate=1:5000) and incubating at room temperature for 50 minutes. Using the newly prepared DAB color developing solution to DAB chromogenic reaction. The color developing time is controlled under the microscope. Rinse the sections with tap water to stop the reaction. Re-staining with hematoxylin for three minutes, hematoxylin differentiation solution differentiates for several seconds, hematoxylin returning blue solution to return blue, water rinsing respectively to make nucleus counterstaining. Different degrees of alcohol for dehydration, xylene for transparency, neutral gum for mounting the sections. The process of the detecting of α-SMA, MYH9, MYH11 is the same as above using the α-SMA antibody (dilution rate=1:5000, 55135-1-AP, Proteintech, Rosemont, USA), MYH9 antibody (dilution rate=1:5000, 11128-1-AP, Proteintech, Rosemont, USA), MYH11 antibody (dilution rate=1:5000, NBP2-59221, Novus, Colorado, USA).

The nucleus was blue and the positive expression of DAB is brownish yellow. Panoramic section scanner [PANNORAMIC 3DHISTECH (Hungary)] is used to scan and image all the tissue information on the tissue section to form a folder which contain all the tissue information. The folder can open with Case Viewer 2.4 software (3DHISTECH (Hungary) and can be enlarged to any multiple of 1-400 times for observation. The Densito Quant module in Quant Center 2.1 analysis software (3DHISTECH, Hungary)is used to quantify the H-score of the target region of each section（H-SCORE=∑（PI×I）= (percentage of cells of weak intensity ×1)+(percentage of cells of moderate intensity ×2)+percentage of cells of strong intensity ×3), the Pi represents the proportion of pixel area of positive signal; I represents the color intensity.

2.2.3.2 Immunofluorescence

Washing three times with PBS (pH7.4) (5min/time), immersed the sections in EDTA antigen retrieval buffer (pH 8.0) (Servicebio G1206, Wuhan, China) to make antigen retrieval. Treat with PBS(PH7.4) (three times, 5min/time), adding 3%BSA（Servicebio, G5001，Wuhan, China）to block non-specific binding for 30min. Throwing away the blocking solution, sections are incubated by MYH10 antibody (dilution rate=1:1000, nbp1-55146, Novus, Colorado, USA) (overnight, at 4℃). Washing the sections again with PBS(PH7.4), fluorescent secondary antibodies (diluteon rate=1:5000) responding to the primary antibodies were added (room temperature, 50min, dark condition). Then incubated with DAPI solution (Servicebio, G1012, Wuhan, China) (room temperature, 10min, darkness) to counterstaining nucleus.

Finally, using spontaneous fluorescence quenching reagent (5min) (Servicebio, G1221, Wuhan, China) to make spontaneous fluorescence quenching and sealing the sections with anti-fade mounting medium. The process of the detecting of α-SMA, MYH9, MYH11 is the same as above using the α-SMA antibody (dilution rate=1:5000, 55135-1-AP, Proteintech, Rosemont, USA), MYH9 antibody (dilution rate=1:5000, 11128-1-AP, Proteintech, Rosemont, USA), MYH11 antibody (dilution rate=1:5000, NBP2-59221, Novus, Colorado, USA). Fluorescence microscopy (Nikon NIKON ECLIPSE C1) showed that the nuclei were blue (excitation wavelength 330-380nm and emission 420nm) and the positive expression was red or green (FITC glows green by excitation wavelength 465-495nm and emission 515-555nm; CY3 glows red by excitation wavelength 510-560nm and emission 590nm)

2.2.3.3 Real-time quantitative polymerase chain reaction (RT-qPCR)

Firstly, putting 1mlRNA extract (Servicebio，G3013，Wuhan, China) and 100mg tissue into homogenate tube. Grinding fully, repeated centrifugation and with the help of chloroform and isopropanol to extract RNA. The primers of the genes were as followed [Sequence (5′–3′)]:

MYH10-hF: CTGGTTCAAGAACAGGGCTCA;

MYH10-hR: AATCTGTCCGATGACTGGTGC;

α-SMA-hF: ACCCTGTTGACTGAGGCACC;

α-SMA-hR: ACCATCTCCAGAGTCCAGCAC;

MYH9-hF: CGAGGACCTGGTTCAAAT;

MYH9-hR: GTGCTGGCTGGGTACTTT;

MYH11-hF: TCCTACCAGTGGCAAACC;

MYH11-hR: CACCCTGTGCTTCATACTCT;

GAPDH-hF: TGAAGGTCGGAGTGAACGGAT;

GAPDH-hR: CGTTCTCAGCCTTGACCGTG.

Using ultra-micro spectrophotometer (NanoDrop2000, Thermo) to detect concentration and purity of RNA. Then reverse transcription is performed: template RNA2μg, Oligo (dT)18 Primer0.5μl, Random Hexamer primer0.5μl and deionized water without ribonuclease that makes the total volume to 15μl. 5min at 65℃, cool quickly and then add: 5×Reaction Buffer 4μl, Servicebio®RT Enzyme Mixa 1μl (Servicebio，G3330，Wuhan, China). Finally, quantitative PCR: 2×qPCR Mix7.5μl, 2.5μM gene primer1.5μL, reverse transcription product2.0μL, ddH2O4.0μl, 3 tubes for each reverse product. Then PCR amplification. Analyzing results with 2^-ΔΔCT^. Using GAPDH as internal control.

2.2.3.4 Western blot

Washing the tissue blocks with cold PBS for 2-3times, and then adding RIPA lysis solution (Servicebio, G2002, Wuhan, China) with 10 times of tissue volume (Add protease inhibitors a few minutes before use) in order to make the tissue homogenization. Centrifugation 10 minutes, collecting the supernatant which is the total protein. The extracted proteins were separated by SDS-polyacrylamide gel electrophoresis (SDS-PAGE). Then transfer the protein to the polyvinylidene fluoride (PVDF) membranes. Then, sealed the membranes with 5% skim milk (made by 0.5%TBST) for 1 hour at room temperature. After that, using GAPDH antibody (dilution rate=1:1000, GB12002, Servicebio, Wuhan, China), MYH10 antibody (dilution rate=1:1000, nbp1-55146, Novus, Colorado, USA), α-SMA antibody (dilution rate=1:5000, 55135-1-AP, Proteintech, Rosemont, USA), MYH9 antibody (dilution rate=1:1000, 11128-1-AP, Proteintech, Rosemont, USA), MYH11 antibody (dilution rate=1:1000, NBP2-59221, Novus, Colorado, USA) to detect the corresponding proteins. GAPDH was used as internal reference. HRP-labeled second antibodies of the corresponding species (diluteon rate = 1:5000) were then incubated at room temperature for 30min.

After incubation, reacting with ECL solution in a darkroom and exposed. The exposed film is developed and fixed with developing and fixing reagents (Servicebio, G2019). Finally, the film is scanned and archived. The color is removed by Photoshop. Alpha software processing system is used to analyze the optical density value of the target strip.

2.2.3.5 Correlation analysis and construction of neural network model

The results are presented as the mean ± standard error of the mean. When two groups were compared, an unpaired Student’s t-test was performed to determine statistical significance. The Spearman and Pearson-rho tests were executed to compare intima-media thickness, the expression of MYH10 and α-SMA for the correlation analysis. The receiver operator characteristic (ROC) curve analysis was used to determine the sensitivity and specificity of MYH10 and α-SMA for predicting AS. Matlab (version 2014a) was performed to accomplish the normalization processing of variable values, network simulation, network training, and network initialization. Two input variables included the expression of MYH10 and the expression of α-SMA. One output variable is intima-media thickness. The cubic spline interpolation algorithm was implemented to analyze the high-risk warning range of AS with the expression of MYH10 and α-SMA. The statistical analyses were conducted using SPSS software, version 24.0 (IBM Corp., Armonk, NY, USA). P<0.05 was considered to indicate a statistically significant difference.

2.2.4 Co-expression analysis between MYH10 and α-SMA

2.2.4.1 Docking study of MYH10 and α-SMA

The PDB ID of MYH10 protein was 4pd3. Swiss-model (<https://swissmodel.expasy.org/>) was used to build model for α-SMA protein. The software PyMol 2.3.4 (DeLano Scientific LLC, https://pymol.org/2/) was used to dehydrate and remove ligand for the receptor protein. MGL Tools software (Molecular Graphics Laboratory, The Scripps Research Institute, http://mgltools.scripps.edu/) was used to hydrogenate the receptor protein and calculate the charge. The appropriate box center and box grid point parameters were set to include the active pocket sites that the ligand might bind to. AutoDock Vina 1.5.6 (http://vina.scripps.edu/) was used for molecular docking of receptor proteins. Twenty conformations were obtained by docking study, and the complex conformation with the best docking score was visualized using PyMol 2.3.4.

2.2.4.2 Co-Immunoprecipitation (IP) assay to detect the interaction between MYH10 and α-SMA

The artery tissues were washed 2-3 times with pre-cooled PBS to remove blood stain, cut into small pieces and placed in homogenate tube. Add 10 times IP lysate (add protease inhibitor within a few minutes before use), and homogenate by machine. The homogenate was transferred to a 1.5mL centrifuge tube and cracked on ice for 30min. The supernatant was collected after centrifugation (12000rpm, 4℃ for 10min). And the protein concentration was determined by BCA method. A small amount of supernatant was denatured for input experiment, that is, western blotting was used to detect target protein.

MYH10 protein (Molecular weight: 229KD) was detected using a MYH10 polyclonal antibody (dilution rate = 1:1000, 19673-1-AP, Proteintech, Rosemont, USA). α-SMA proteins (Molecular weight: 43KD) were detected using an α-SMA polyclonal antibody (dilution rate = 1:1000, 14395-1-AP, Proteintech, Rosemont, USA). A secondary antibody was applied (Mouse Anti-Rabbit IgG Light Chain Specific, HRP conjugate, Catalog number: SA00001-7L, dilution rate = 1:3000).

Add 1.0μg IgG and 20μL Protein A/G beads into the negative control (IgG) group supernatant (mix well before use). Add 20μL Protein A/G beads directly into the experimental group, shake and incubate at 4℃ for 1h. The supernatant was extracted after centrifugation at 4℃ for 5min at 2000rpm. Add 1-10 μL (0.2-2μg) antibody, and incubate overnight at 4℃. Add 80μL Protein A/ G-beads (well mixed before use), and incubate at 4℃ for 2h. After centrifugation at 4℃ for 5min at 2000rpm, the supernatant was carefully aspirated and the immunoprecipitation complex was collected. The immunoprecipitation complex was washed 4 times with 1 mL pre-cooled IP lysate (without adding various inhibitors). Centrifuged at 4℃, 2000rpm, for 5min, and carefully discarded the supernatant after each washing. After the last washing, absorb as much supernatant as possible. Then, add 80μl 1× reduced sample loading buffer and boil for 10min.The supernatant was centrifuged at 1000rpm at 4℃ for 5min.Take 10μl supernatant sample for western blotting test.

2.2.5 Co-expression analysis between MYH11 and α-SMA

MYH111 protein (Molecular weight: 33KD) was detected using a MYH11 polyclonal antibody (dilution rate = 1:1000, NBP2-59221, Novus, Colorado, USA). α-SMA proteins (Molecular weight: 43KD) were detected using an α-SMA polyclonal antibody (dilution rate = 1:1000, 14395-1-AP, Proteintech, Rosemont, USA). The process of CO-IP between MYH11 and α-SMA was same as the above.

**3. Results:**

3.1 Significant role of MYH10 on the AS via bioinformatics

3.1.1 Evaluation microenvironment score

In this study, stromal score, immune score and estimate score were evaluated in GSE43292 (Figure S 1A). The area under curve (AUC) of stromal score for AS was 0.772 (P <0.001), and AUC of immune score for AS was 0.833 (P<0.001), estimate score 0.827 (P<0.001) (Figure S 1B).

3.1.2 Detection of different expressed genes (DEGs)

In this study, DEGs were identified based on the level of stromal score in GSE43292, which comprised 3727 up-regulated and 4511 down-regulated DEGs (Figure S 1C). The 9305 DEGs were detected based on the level of immune score, which comprised 4436 up-regulated and 4869 down-regulated DEGs (Figure S 1D). And 6723 different expressed genes (DEGs) were identified based on the status of samples, which comprised 3237 up-regulated and 3486 down-regulated DEGs (Figure S 1E).

3.1.3 WGCNA analysis

The selection of the soft-thresholding power was an important step in WGCNA analysis. Network topology analysis was performed to identify the soft-thresholding power. The soft-thresholding power in the WGCNA analysis was set to 17, which was the lowest power for the scale-free topology fit index of 0.9 (Figure S 2A). A hierarchical clustering tree of all genes were constructed and 14 important modules were generated (Figure S 2B). The dendrogram and heatmap of genes showed that there was no significant difference in interactions among different modules, demonstrating that there was a high degree of independence between these modules (Figure S 2C). The blue module had the highest negative correlation with the status of AS (Figure S 2D). Interactions between these modules were then analyzed (Figure S 2E). The predicted interconnection of genes in the blue module was imported into cytoscape software to search for predicted key genes. A total of 118 predicted key genes were derived through four algorithms (Closeness, MCC, MNC, Radiality) (Figure S 2F).

3.1.4 Identification of key DEGs

Furthermore, in this study, we identified 118 predicted key genes in the blue module, which were differentially expressed in the three types of groups (DEGs identified by stromal score, DEGs identified by immue score, DEGs identified by status of disease) (Figure S 2G).

3.1.5 Construction and analysis of protein-protein interaction (PPI) network

The PPI networks of 118 predicted key DEGs were constructed by STRING online database and analyzed by Cytoscape software (Figure S 2H). Four different algorithms were employed to identify hub genes and 4 common hub genes were obtained, and MYH10 was the one hub genes of AS (Figure S 2I).

3.1.6 Functional enrichment analysis

In this study, GO and KEGG analyses of blue module were performed, which may improve the biological understanding of these genes. As shown in Figure S 3A that according to GO analysis, they were mainly enriched in the blood vessel morphogenesis, contractile fiber part, chemotaxis, regulation of MAPK cascade. The results of KEGG analysis showed that target genes were mainly enriched in MAPK signaling pathway, fluid shear stress and atherosclerosis, cytokine-cytokine receptor interaction (Figure S 3B). The P values of the GO terms are shown in Figure S 3C. The P value of KEGG terms is shown in Figure S 3D.

3.1.7 Single gene analysis of MYH10

Compared with normal tissues, the expression of MYH10 was lower in the AS tissue (P<0.05). MYH10 was differentially expressed in GSE43292 (Figure S 4A) and also paired differentially expressed (Figure S 4B). Association between MYH10 and AS was analyzed by ROC curve, and the AUC is 0.813 (P<0.001) (Figure S 4C). In our study, we grouped the samples according to the level of expression of MYH10, and the GO and KEGG analysis of the complete genome were performed by GSEA, which showed that the main enriched terms included contractile fiber, myosin filament, structure constituent of muscle, ubiquitin protein transferase regulator activity. (Figure S 4D).

3.1.8 Evaluation of AS-inﬁltrating immune cells

In the AS-inﬁltrating immune cell matrix, the stacked histogram shows the proportion of immune cells in each sample (Figure S 5A) and heatmap revelated the content of immune cells in each sample (Figure S 5B). Correlations among 22 immune cells were showed in Figure S 5C. As shown in Figure S 5D-5E, monocytes, T cells CD4 memory activated, B cells naive, dendritic cells resting, macrophages M0, NK cells activated, B cells memory and T cells CD8 infiltration content were different (P<0.05).

3.2 Absence of MYH10 in the AS compared with the normal tissue through animal model

3.2.1 Successful construction of the AS-animal model

The HE staining (30x, 200x, 400x) results showed that the intima of the arterial sections in the control group was smooth and orderly, without bulge, thickened intima membrane, or lipid deposition (Figure S 6A). However, in the AS-animal model, the intima of the arterial section was damaged and disorganized, with obviously thickened intima membrane (Figure S 6B). Through quantitative analysis, intima-media thickness in the AS-animal model group was significantly thicker than the control group (P<0.001, Figure S 6C).

3.2.2 Expression of MYH10 via the immunohistochemical and immunofluorescence assay

The immunohistochemical assay (30x, 200x, 400x) results showed that the MYH10 was clearly expressed in the media of the arteries, and there were very few expressions in the intima membrane (The brown-yellow color represents the expression of the MYH10 molecule, and the blue color represents the nucleus) (Figure S 7A). However, in the AS-animal model, the MYH10 was down-expressed significantly in the media of arteries (Figure S 7B).

Furthermore, the immunofluorescence assay also manifested that compared with the control group, the expression of MYH10 in the media of the arteries was lower in the AS-animal model group, and there were very few expressions in the intima membrane (The red color represents the expression of the MYH10 molecule, and the blue color represents the nucleus) (Figure S 7C).

3.2.3 Expression of α-SMA via the immunohistochemical and immunofluorescence assay

The immunohistochemical assay (30x, 200x, 400x) results showed that the α-SMA was clearly expressed in the media of the arteries, and there were few expressions in the intima membrane (The brown-yellow color represents the expression of the α-SMA molecule, and the blue color represents the nucleus) (Figure S 8A). However, in the AS-animal model, the α-SMA was down-expressed significantly in the media of arteries (Figure S 8B).

Furthermore, the immunofluorescence assay also manifested that compared with the control group, the expression of α-SMA in the media of the arteries was higher in the AS-animal model group, and there were very few expressions in the intima membrane (The red color represents the expression of the α-SMA molecule, and the blue color represents the nucleus) (Figure S 8C).

3.2.4 The expression of MYH10 and α-SMA, and strong associations among MYH10, α-SMA, and IMT

Compared with the control group, the expressions of MYH10 and α-SMA in the mRNA level were down-expressed in the AS-animal group significantly (Figure S 9A). The IMT was negatively related to the expression of MYH10 (Spearman Rho=-0.606, P=0.005), and the α-SMA expression was also negatively associated with the IMT (Spearman Rho=-0.671, P=0.001). And there is a positive association between MYH10 and α-SMA expression (Spearman Rho=0.810, P<0.001) (Figure S 9B, C). In the Western blotting assay, the expressions of MYH10 and α-SMA were lower in the AS than the control group (Figure S 9D). The results of ROC demonstrated that MYH10 expression could specifically and sensitively predict IMT [area under the curve (AUC) = 0.980; P<0.001]. In addition, the α-SMA expression also play a significant role in the development of IMT (P<0.001) (Figure S 9E).

3.2.5 Prediction of IMT based on the neural network model and cubic spline interpolation algorithm

Through the training of neural network model, the best training performance is 0.035651 at epoch 3000 (Figure S 10A). The relativity of the model (built by MYH10, α-SMA and IMT) is 0.94383 (Figure S 10B). Furthermore, the model could be verified well, and there exists tiny error between raw data and forecast data (Figure S 10C, D). The above result could demonstrate that the joint effect of MYH10 and α-SMA would be predictive parameters of IMT in the AS. Results of cubic spline interpolation algorithm showed that the high-risk warning indicator of IMT of the AS: 0.90 < MYH10 < 2.70, and 0 < α-SMA < 0.90 (Figure S 10E, F).

3.3 Verification for the role of the MYH10 on AS via clinical samples

3.3.1 Based characteristics of patients and the expression of MYH10

Among the samples, the group with low expression of MYH10 consisted of 16(47.1%) male and 5(14.7%) female, and the group with high expression of MYH10 consisted of 11(32.4%) male and 2(5.9%) female. In terms of the age, the group with low expression of MYH10 consisted of 3 (8.8%) samples of years old <60 and 18(52.9%) samples of years old ≥60, and the group with high expression of MYH10 consisted of 1(2.9%) samples of years old <60 and 12(35.3%) samples of years old ≥60. There were no differences between the low and high MYH10 expression in terms of diabetes, hypertension, smoking, drinking, hematencephalon, cerebral infarction, coronary heart disease (P>0.05). The average and standard deviation of IMT was 3593.51±1502.64 μm in the group with low MYH10 expression, and 1809.73±1231.81μm in the group with high MYH10 expression (P<0.001). The relative expression H-score of α-SMA was 82.59±37.96 in the low MYH10 expression group, and 114.33±13.19 in the high MYH10 expression group (P=0.009) (Table 1).

3.3.2 Relationship between characteristics of patients and MYH10 via Spearman correlation test

The expression of MYH10 was significantly related to IMT (ρ=-0.514, P=0.002), atherosclerosis (ρ=-0.525, P=0.001), and α-SMA (ρ=0.3688, P=0.032). However, the other characteristics were not correlated to the expression of MYH10 (Table 2).

3.3.3 Advantage of high expression of MYH10 and α-SMA on the atherosclerosis

The odds ratio (OR) for atherosclerosis was 0.073 (95% CI, 0.012-0.431, P=0.004) in the group with high MYH10 expression levels compared with that in the group with low MYH10 expression group. It can be seen that sex&MYH10 (OR=0.147, 95% CI: 0.033-0.659, P=0.012), age&MYH10 (OR=0.357, 95%CI: 0.162-0.788, P=0.011), smoking&MYH10 (OR=0.181, 95%CI: 0.041-0.793, P=0.023), drinking&MYH10 (OR=0.117, 95%CI: 0.024-0.576, P=0.008), hematencephalon&MYH10 (OR=0.106, 95%CI: 0.021-0.536, P=0.007), α-SMA&MYH10 (OR=0.316, 95%CI: 0.148-0.674, P=0.003) have clear correlations with atherosclerosis. However, there were no significant correlations between atherosclerosis and between diabetes&MYH10, hypertension&MYH10, cerebral infarction&MYH10 (Table 3).

3.3.4 Molecular docking study of MYH10 and α-SMA

The binding pattern between the ligand and the receptor protein was studied by molecular docking. Binding energy of docking conformations between MYH10 and α-SMA was calculated, and docking score between the MYH10 and α-SMA was -7.29 kcal/mol (Table 4). The lowest binding energy of optimal complex conformation was -7.29 kcal/mol. Hydrogen bond interaction exists between the amino acid residues in MYH10 (LYS-78, GLY-773, GLN-77, ALA-63) and α-SMA (PRO-29, ARG-30, TRP-37, ALA-47) (Table 5).

3.4 Gradual changes of intima-media thickness (IMT) in pathology, and the expression of α-SMA, MYH10.

With the passage of time, the IMT gradually increased. However, with the passage of time, the expression of α-SMA and MYH10 gradually decreased (Figure S11A). The HE staining showed the gradual changes of IMT in pathology, and the IMT was thicker in the fourth week than the previous artery (Figure S11B). There existed negative correlations between IMT and expression of α-SMA (and MYH10). However, the expression of MYH10 was positively related with the α-SMA (Figure S11C).

3.5 The expression of MYH11 and MYH9 in the control artery and atherosclerosis samples in the animal model.

Through the immunohistochemical assay, the expression of MYH11 in control artery samples was up-regulated compared with the atherosclerosis samples (P<0.001, Figure S12A, B). And the RT-qPCR also verified that the expression of MYH11 in the atherosclerosis samples was lower than the control artery samples (P<0.001, Figure S12C). Through the RT-qPCR assay, the expression of MYH9 in control artery samples was up-regulated compared with the atherosclerosis samples (P<0.001, Figure S12D). The relative expression of MYH9 in the artery in animal model was negatively related with the intima-media thickness (R=-0.919, P<0.001, Figure S12E). Through the immunohistochemical assay, the expression of MYH9 in atherosclerosis samples was lower than the control artery samples (P<0.001, Figure S12F).

3.6 Verification for the role of MYH11 based on the clinical AS samples.

The immunohistochemical assay (100x, 200x, 400x) results showed that the MYH11 was clearly expressed in the media of the arteries, and there were few expressions in the intima membrane in the normal group (The brown-yellow color represents the expression of the MYH11 molecule, and the blue color represents the nucleus). However, in the clinical AS samples, the MYH11 was down-expressed significantly in the media of arteries (Figure S13A). Furthermore, the immunofluorescence assay also manifested that compared with the normal group, the expression of MYH11 in the media of the arteries was higher in the clinical AS group, and there were very few expressions in the intima membrane (The red color represents the expression of the MYH11 molecule, and the blue color represents the nucleus) (Figure S13B). Quantitative analysis of expression of MYH11 IHC-Score manifested that the expression of MYH11 was significantly lower in the clinical AS tissues than in the control group (P=0.006, Figure S13C). Quantitative analysis of relative protein expression of MYH11 in clinical sample via immunofluorescence assay between the control and AS samples (P<0.001, Figure S13D). The RT-PCR results indicated that the relative expression level of MYH11 mRNA was significantly decreased in clinical atherosclerosis artery compared with control artery tissue samples without atherosclerosis (P=0.009, Figure S13E). Western blotting analysis showed that the expression of MYH11 proteins was down-regulated in the clinical AS samples compared with the control group (P=0.045, Figure S13F, G).

3.7 Verification for the role of MYH9 based on the clinical AS samples.

The immunohistochemical assay (100x, 200x, 400x) results showed that the MYH9 was clearly expressed in the media of the arteries, and there were few expressions in the intima membrane in the normal group (The brown-yellow color represents the expression of the MYH9 molecule, and the blue color represents the nucleus). However, in the clinical AS samples, the MYH9 was down-expressed significantly in the media of arteries (Figure S14A). Furthermore, the immunofluorescence assay also manifested that compared with the normal group, the expression of MYH9 in the media of the arteries was higher in the clinical AS group, and there were very few expressions in the intima membrane (The red color represents the expression of the MYH9 molecule, and the blue color represents the nucleus) (Figure S14B). Quantitative analysis of expression of MYH9 IHC-Score manifested that the expression of MYH9 was significantly lower in the clinical AS tissues than in the control group (P=0.006, Figure S14C). Quantitative analysis of relative protein expression of MYH9 in clinical sample via immunofluorescence assay between the control and AS samples (P<0.001, Figure S14D). The RT-PCR results indicated that the relative expression level of MYH9 mRNA was significantly decreased in clinical atherosclerosis artery compared with control artery tissue samples without atherosclerosis (P=0.009, Figure S14E). Western blotting analysis showed that the expression of MYH9 proteins was down-regulated in the clinical AS samples compared with the control group (P=0.045, Figure S14F, G).

3.8 Co-expression analysis between MYH11 and α-SMA.

Through the Co-Immunoprecipitation assay, the MYH11 and α-SMA were co-expressed in the artery (Figure S15).

**4. Discussion:**

MYH10 gene encodes non-muscular myosin ⅡB, which plays a fundamental role in the maintenance of cell morphology, cell adhesion, migration and cell division^5^. Wang et al. found that MYH10 was involved in cell migration, invasion, production of extracellular matrix (ECM) and epithelial-mesenchymal transformation. Taskinen et al. found that MYH10 plays a new role in cancer progression by regulating cell movement and actin contraction through MASTL^6^. Antony et al. demonstrated through experiments that MYH10 detection can be used as a new simple tool to identify diseases with abnormalities in RUNX1 and its related proteins^7^. Meanwhile, MYH10 is one of the markers of the proliferative phenotype of vascular smooth muscle cells (VSMCs)^8^. A large number of studies have confirmed that VSMCs are one of the important markers of atherosclerosis (AS) and play a very important role in AS^9^. In this study, it was found that the expression of MYH10 molecule was positively correlated with the number of vascular smooth muscle cells.

Some scholars believe that phenotypic transformation of VSMCs plays an important role in the formation of AS, and inhibition of phenotypic transformation of VSMCs has a protective effect on arteries. VSMCs in arterial media normally express smooth muscle cell markers such as MYH11, MYH10, smooth muscle 22α (SM22α), ACTA2, and smooth muscle cell differentiation specific antigen (SMTN), but the ability of VSMCs in arterial media with atherosclerosis to express these markers decreases. Current studies have found that phenotypic transformation of VSMCs can occur under both physiological and pathological conditions, mainly differentiation into macrophages, and phenotypic transformation of VSMCs has the markers and characteristics of macrophages. Macrophages are the most important and abundant immune cells in the damage process of AS. Macrophages play an important role in every link from the onset of AS damage to the rupture of AS plaque. The researchers believe that macrophages from blood monocytes are precursors of lipid foam cells in atherosclerosis. Recent experimental data suggest that smooth muscle cell metaplasia may also produce foam cells similar to macrophages.

Foam cells are mainly derived from macrophages and VSMCs, among which the foam cells derived from VSMCs are called smooth muscle derived foam cells. Smooth muscle-derived foam cells are common in the early and advanced plaques of human AS, so smooth muscle-derived foam cells are one of the important markers of the formation of AS. Brown et al.^10^ showed tha once the smooth muscle-derived foam cells are formed, a series of reactions will occur. For example, the accumulation of cholesterol will induce the apoptosis of VSMCs and promote the migration of neighboring VSMCs to the intima. The apoptosis or death of VSMCs will also aggravate the inflammatory response.Meanwhile, chemokine C-C ligand 19 (CCL19) can directly regulate phenotypic transformation, growth and release of MMPs in VSMCs, and these pathophysiological processes can promote intima thickening and AS formation^11^. She et al.^12^ have shown that extracellular matrix can inhibit phenotypic transformation of VSMCs, while matrix metalloproteinase (MMP) released by macrophages or VSMCs can lead to the dissociation of extracellular matrix, collagen or elastic fibers. Thus, phenotypic transformation of VSMCs was promoted and cell proliferation and migration were accelerated. Studies by Rohwedder et al.^13^ showed that the deposition of adhesin can promote the formation of AS, but it can also promote the formation of fibrous cap, thus enhancing the stability of AS plaque. However, there are differences between animal models and human bodies. For example, there are fewer smooth muscle-derived foam cells in AS plaques of animals with hypercholesterolemia, but more smooth muscle-derived foam cells in the advanced stage of AS^14^. Previous studies have shown that ATP-binding cassette transporter A1 (ABCA1) and apolipoprotein A1 (APO A1) are highly expressed in arterial intima VSMCs. ABCA1 and Apo A1 play an important role in cholesterol transport by mediating the outflow of cholesterol from the cell to form high-density lipoprotein (HDL)^10^. Therefore, the imbalance of lipid uptake and outflow in the early stage of AS formation leads to the foaming of V reversal SMCs.

About 40% of foam cells in human coronary artery progression expressed both the smooth muscle cell marker actin α2 and the macrophage marker CD68^15^. As an important part of phenotypic transformation of VSMCs, the cardiomycin/serum response factor regulatory model can effectively integrate signals and cofactors that activate and/or inhibit VSMCs contraction genes^16^. In addition, studies by Allahverdlan et al.^17^ showed that vascular smooth muscle cultured in cholesterol medium activates various pro-inflammatory factors through Kruppel like factor 4 (KLF4), inhibits the expression of VSMCs markers, activates macrophage markers and induces phagocytosis. It is suggested that lipid accumulation in atherosclerotic plaques may lead to the transformation of VSMCs into macrophage-like cell phenotypes. Compared with monocytes, macrophages and dendritic cells, the phagocytic ability of smooth muscle-derived macrophages was significantly reduced, and the phagocytic ability (such as the phagocytic ability of apoptotic cells) was significantly decreased during the progression of atherosclerosis. Therefore, phenotypic transformation of VSMCs may be involved in the progression of atherosclerosis.

In addition, our study found that the expression of MYH10 molecule and the number of smooth muscle cells were negatively correlated with the thickness of intravascular media. The middle layer of vascular wall is mainly composed of VSMCs, which are one of the important cells in the regulation of vascular blood pressure and play an important role in the formation of a variety of vascular diseases such as atherosclerosis, vascular restenosis and hypertension. The apoptosis rate of atherosclerosis cells is low in the early stage, but gradually increases with the development of atherosclerosis with the formation of necrotic core and fibrous cap, among which macrophages and VSMCs are the cells with the highest apoptosis rate. Plaques rupture mainly occurred in the shoulder of the plaque, which was characterized by decreased VSMCs and increased macrophages, etc. The reason might be that macrophages induced apoptosis of VSMCs through the interaction of death receptors and death ligands, which played an important role in plaque rupture and cardiovascular events. In addition, the rate of VSMCs apoptosis was higher in unstable plaques than in stable plaques^18^. Previous studies have shown that the apoptosis of VSMCs in AS patients is related to inflammation, but the inflammatory response is significantly weakened during vascular aging, matrix degradation and vascular remodeling, and the reason may be related to the release of cytokines during the clearance of apoptotic cells. Interleukin 1 (IL-1) is released during cell death, interleukin 1β (IL-1β) and interleukin 1α (IL-1α) are released during cell apoptosis and necrosis, respectively, and both interleukin 1β and IL-1α are released during cell necrosis following apoptosis. Apoptotic cells are generally cleared within 48 h, but the phagocytosis process is delayed in hyperlipidemia, so the inflammatory response after phenotypic transformation of VSMCs is associated with reduced phagocytosis. In addition, a genome highly associated with cardiovascular disease, region 2 band 1 (9p21) on the long arm of human chromosome 9, is strongly associated with reduced expression of cyclin-dependent kinase inhibitor 2B and calcium reticulin, which is a necessary ligand for activation of phagocytic receptors on phagocytes^19^. Apoptotic bodies with cyclin-dependent kinase inhibitor 2B deficiency are anti-phagocytic and cannot be effectively phagocyzed by neighboring macrophages. Previous studies have shown that impaired cell interment capacity caused by cyclin-dependent kinase inhibitor 2B deficiency increases the area and complexity of the necrotic core under lipid load, thereby exacerbating atherosclerosis^20-22^.

Although this study conducted rigorous bioinformatics analysis, and used animal experiments, human specimen experiments and cell function experiments to rigorously verify the role of MYH10 and surface changes of smooth muscle cells in atherosclerosis. However, this paper still has some shortcomings. First of all, this study did not conduct a multicenter randomized controlled clinical trial on MYH10 and α-SMA to verify their important value in atherosclerosis. Secondly, due to the COVID-19 pandemic, it is difficult to obtain clinical atherosclerosis specimens, resulting in a relatively small amount of data included in this paper. However, we will further explore this in future research.

**REFERENCES**

1. Ahmadi A, Argulian E, Leipsic J, Newby DE, Narula J. From Subclinical Atherosclerosis to Plaque Progression and Acute Coronary Events: JACC State-of-the-Art Review. *J Am Coll Cardiol*. 2019;74(12):1608-1617.

2. Martínez GJ, Celermajer DS, Patel S. The NLRP3 inflammasome and the emerging role of colchicine to inhibit atherosclerosis-associated inflammation. *Atherosclerosis*. 2018;269:262-271.

3. Mathers CD, Loncar D. Projections of global mortality and burden of disease from 2002 to 2030. *PLoS Med*. 2006;3(11):e442.

4. Meng LB, Shan MJ, Qiu Y, et al. TPM2 as a potential predictive biomarker for atherosclerosis. *Aging (Albany NY)*. 2019;11(17):6960-6982.

5. Newell-Litwa KA, Horwitz R, Lamers ML. Non-muscle myosin II in disease: mechanisms and therapeutic opportunities. *Dis Model Mech*. 2015;8(12):1495-1515.

6. Taskinen ME, Närvä E, Conway J, et al. MASTL promotes cell contractility and motility through kinase-independent signaling. *J Cell Biol*. 2020;219(6).

7. Antony-Debré I, Bluteau D, Itzykson R, et al. MYH10 protein expression in platelets as a biomarker of RUNX1 and FLI1 alterations. *Blood*. 2012;120(13):2719-2722.

8. Song JB, Shen J, Fan J, et al. Effects of a Matrix Metalloproteinase Inhibitor-Eluting Stent on In-Stent Restenosis. *Med Sci Monit*. 2020;26:e922556.

9. Deng Y, Zhou Z, Lin S, Yu B. METTL1 limits differentiation and functioning of EPCs derived from human-induced pluripotent stem cells through a MAPK/ERK pathway. *Biochem Biophys Res Commun*. 2020;527(3):791-798.

10. Brown BA, Williams H, George SJ. Evidence for the Involvement of Matrix-Degrading Metalloproteinases (MMPs) in Atherosclerosis. *Prog Mol Biol Transl Sci*. 2017;147:197-237.

11. Pfaltzgraff ER, Bader DM. Heterogeneity in vascular smooth muscle cell embryonic origin in relation to adult structure, physiology, and disease. *Dev Dyn*. 2015;244(3):410-416.

12. She ZG, Chang Y, Pang HB, et al. NG2 Proteoglycan Ablation Reduces Foam Cell Formation and Atherogenesis via Decreased Low-Density Lipoprotein Retention by Synthetic Smooth Muscle Cells. *Arterioscler Thromb Vasc Biol*. 2016;36(1):49-59.

13. Rohwedder I, Montanez E, Beckmann K, et al. Plasma fibronectin deficiency impedes atherosclerosis progression and fibrous cap formation. *EMBO Mol Med*. 2012;4(7):564-576.

14. Zamanian-Daryoush M, Lindner DJ, DiDonato JA, et al. Myeloid-specific genetic ablation of ATP-binding cassette transporter ABCA1 is protective against cancer. *Oncotarget*. 2017;8(42):71965-71980.

15. Yeung KK, Bogunovic N, Keekstra N, et al. Transdifferentiation of Human Dermal Fibroblasts to Smooth Muscle-Like Cells to Study the Effect of MYH11 and ACTA2 Mutations in Aortic Aneurysms. *Hum Mutat*. 2017;38(4):439-450.

16. Alexander MR, Owens GK. Epigenetic control of smooth muscle cell differentiation and phenotypic switching in vascular development and disease. *Annu Rev Physiol*. 2012;74:13-40.

17. Allahverdian S, Chehroudi AC, McManus BM, Abraham T, Francis GA. Contribution of intimal smooth muscle cells to cholesterol accumulation and macrophage-like cells in human atherosclerosis. *Circulation*. 2014;129(15):1551-1559.

18. Gonzalez L, Trigatti BL. Macrophage Apoptosis and Necrotic Core Development in Atherosclerosis: A Rapidly Advancing Field with Clinical Relevance to Imaging and Therapy. *Can J Cardiol*. 2017;33(3):303-312.

19. Kojima Y, Downing K, Kundu R, et al. Cyclin-dependent kinase inhibitor 2B regulates efferocytosis and atherosclerosis. *J Clin Invest*. 2019;129(5):2164.

20. Abu-Amero KK, Kondkar AA, Mousa A, et al. Analysis of Cyclin-Dependent Kinase Inhibitor-2B rs1063192 Polymorphism in Saudi Patients with Primary Open-Angle Glaucoma. *Genet Test Mol Biomarkers*. 2016;20(10):637-641.

21. Gao P, Si J, Yang B, Yu J. Upregulation of MicroRNA-15a Contributes to Pathogenesis of Abdominal Aortic Aneurysm (AAA) by Modulating the Expression of Cyclin-Dependent Kinase Inhibitor 2B (CDKN2B). *Med Sci Monit*. 2017;23:881-888.

22. Yu JH, Zhu BM, Wickre M, et al. The transcription factors signal transducer and activator of transcription 5A (STAT5A) and STAT5B negatively regulate cell proliferation through the activation of cyclin-dependent kinase inhibitor 2b (Cdkn2b) and Cdkn1a expression. *Hepatology*. 2010;52(5):1808-1818.

| Table 1. Based characteristics of patients and the expression of MYH10 | | | | |
| --- | --- | --- | --- | --- |
| Characteristics | | MYH10 | | P |
|  |  | Low (%) | High (%) |  |
| Sex |  |  | | 0.682 |
| Male | 27 | 16(47.1%) | 11(32.4%) |  |
| Female | 7 | 5(14.7%) | 2(5.9%) |  |
| Age |  |  | | 0.647 |
| <60 | 4 | 3(8.8%) | 1(2.9%) |  |
| ≥60 | 30 | 18(52.9%) | 12(35.3%) |  |
| Diabetes |  |  | | 0.427 |
| No | 25 | 14(41.2%) | 11(32.4%) |  |
| Yes | 9 | 7(20.6%) | 2(5.9%) |  |
| Hypertension |  |  | | 0.709 |
| No | 11 | 6(17.6%) | 5(14.7%) |  |
| Yes | 23 | 15(44.1%) | 8(23.5%) |  |
| Smoking |  |  | | 0.855 |
| No | 31 | 19(55.9%) | 12(35.3%) |  |
| Yes | 3 | 2(5.9%) | 1(2.9%) |  |
| Drinking |  |  |  |  |
| No | 33 | 20(58.8%) | 13(38.2%) | 0.425 |
| Yes | 1 | 1(2.9%) | 0(0.0%) |  |
| Hematencephalon |  |  |  |  |
| No | 30 | 19(55.9%) | 11(32.4%) | 0.606 |
| Yes | 4 | 2(5.9%) | 2(5.9%) |  |
| Cerebral infarction |  |  |  |  |
| No | 19 | 12(35.3%) | 7(20.6%) | 0.851 |
| Yes | 15 | 9(26.5%) | 6(17.6%) |  |
| CHD |  |  |  |  |
| No | 24 | 15(44.1%) | 9(26.5%) | 0.891 |
| Yes | 10 | 6(17.6%) | 4(11.8%) |  |
| Atherosclerosis |  |  |  |  |
| No | 17 | 6(17.6%) | 11(32.4%) | 0.004* |
| Yes | 17 | 15(44.1%) | 2(5.9%) |  |
| IMT | 2911.47±1641.46 | 3593.51±1502.64 | 1809.73±1231.81 | 0.001* |
| α-SMA | 94.73±34.38 | 82.59±37.96 | 114.33±13.19 | 0.002* |
| Fisher test was used for categorical variable. For continuous variable, independent-samples T test was used, and when the equal variances not assumed, Brown-Forsythe was performed. CHD: Coronary heart disease. IMT: intima-media thickness; MYH10: Myosin Heavy Chain 10; α-SMA: α-smooth muscle actin. *P<0.05. | | | | |

| Table 2. The relationship between characteristics of patients and MYH10 | | |
| --- | --- | --- |
| Characteristics | MYH10 | |
|  | ρ | P |
| Sex | -0.063 | 0.723 |
| Age | -0.065 | 0.714 |
| Diabetes | -0.160 | 0.367 |
| Hypertension | -0.125 | 0.481 |
| Smoking | 0.005 | 0.976 |
| Drinking | -0.204 | 0.247 |
| Hematencephalon | -0.009 | 0.958 |
| Cerebral infarction | 0.006 | 0.973 |
| CHD | -0.053 | 0.767 |
| IMT | -0.514 | 0.002* |
| Atherosclerosis | -0.525 | 0.001* |
| α-SMA | 0.388 | 0.023* |
| Spearman correlation test was used. CHD: Coronary heart disease. IMT: intima-media thickness; MYH10: Myosin Heavy Chain 10; α-SMA: α-smooth muscle actin. *P<0.05. | | |

| Table 3. Effect of MYH10 on atherosclerosis based on univariate logistic proportional regression analysis | | | | | |
| --- | --- | --- | --- | --- | --- |
| Parameters | | | Atherosclerosis | | |
|  |  |  | OR | 95% CI | P |
| MYH10 | Low | 21 | 1 |  | 0.004* |
|  | High | 13 | 0.073 | 0.012-0.431 |  |
| Sex&MYH10 |  |  | 0.147 | 0.033-0.659 | 0.012* |
| Age&MYH10 |  |  | 0.357 | 0.162-0.788 | 0.011* |
| Diabetes&MYH10 |  |  | 0.495 | 0.173-1.421 | 0.191 |
| Hypertension&MYH10 |  |  | 0.541 | 0.258-1.135 | 0.104 |
| Smoking&MYH10 |  |  | 0.181 | 0.041-0.793 | 0.023* |
| Drinking&MYH10 |  |  | 0.117 | 0.024-0.576 | 0.008* |
| Hematencephalon&MYH10 |  |  | 0.106 | 0.021-0.536 | 0.007* |
| Cerebral infarction&MYH10 |  |  | 0.546 | 0.259-1.150 | 0.111 |
| α-SMA&MYH10 |  |  | 0.316 | 0.148-0.674 | 0.003* |
| OR, odds ratio; 95% CI, 95% confidence interval. MYH10: Myosin Heavy Chain 10; α-SMA: α-smooth muscle actin. *P<0.05. | | | | | |

| Table 4. The docking score between MYH10 and α-SMA | | |
| --- | --- | --- |
| Protein | Grid_size | Docking score (kcal/mol) |
|  |  | α-SMA |
| MYH10 | 66×66×112 | -7.29 |

| Table 5. Hydrogen bond interaction between the amino acid residues in MYH10 and α-SMA | | | |
| --- | --- | --- | --- |
|  | α-SMA | MYH10 | Distance (A) |
| Docking Scheme 1 | PRO-29 | LYS-78 | 2.6 |
|  | ARG-30 | GLY-773 | 3.2 |
|  | TRP-37 | GLN-77 | 2.1 |
|  | ALA-47 | ALA-63 | 2.7 |


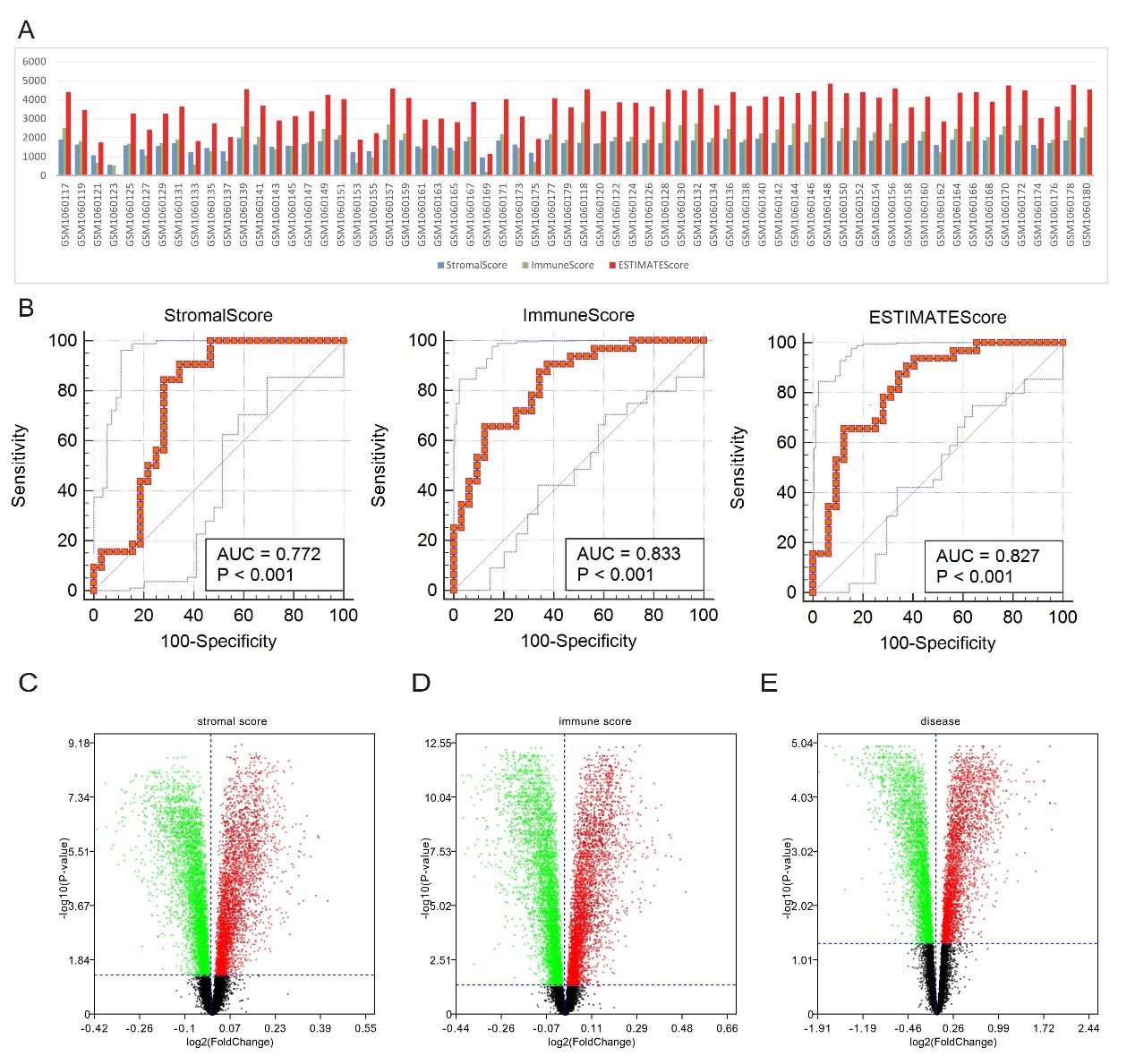


Figure S1. Microenvironment in AS. (A) The histogram shows the microenvironment score (stromal score, immune score, ESTIMATE score). (B) ROC curve shows the relationship between the microenvironment scores and AS. (C) Volcano plot of the genes showing differential expression (DEGs) between high stromal score group and low stromal score group. (D) Volcano plot of DEGs between high immune score group and low immune score group. (E) Volcano plot of DEGs between AS group and normal group.


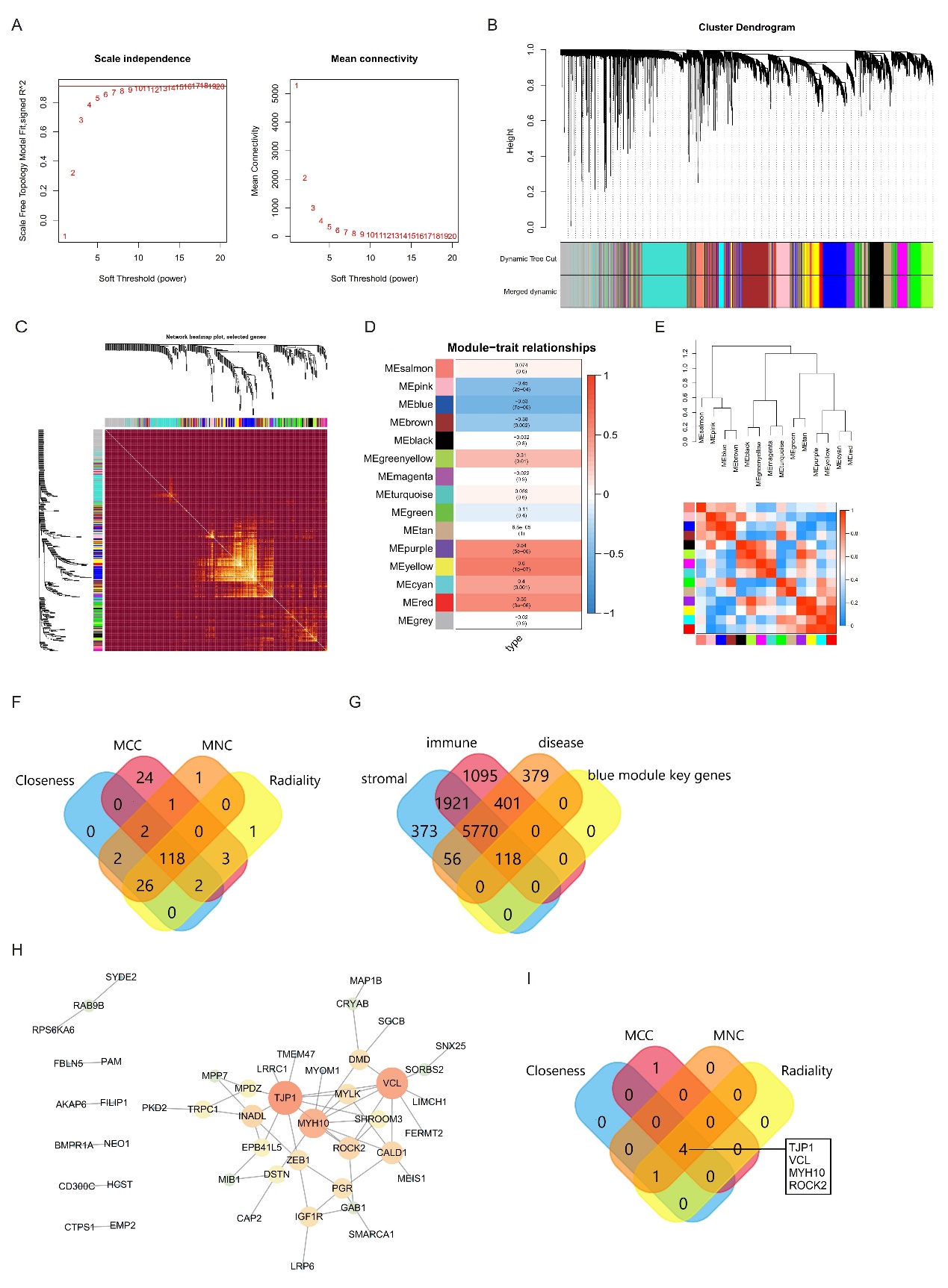


Figure S2. WGCNA analyses and hub genes in GSE43292. (A) The lowest power for which scale independence. (B) Repeated hierarchical clustering tree of all genes. (C) The dendrogram and heatmap of all genes. (D) The associations between clinical traits and the modules. (E) Interactions between these modules. (F) The common key genes identified from the predicted interaction network in blue module by different algorithm. (G) Venn diagrams showing that the common key genes are the common DEGs in three grouping methods. (H) Protein-protein interaction (PPI) network, the more the number of connections, the larger of the protein. (I) The common hub genes identified from different algorithm.


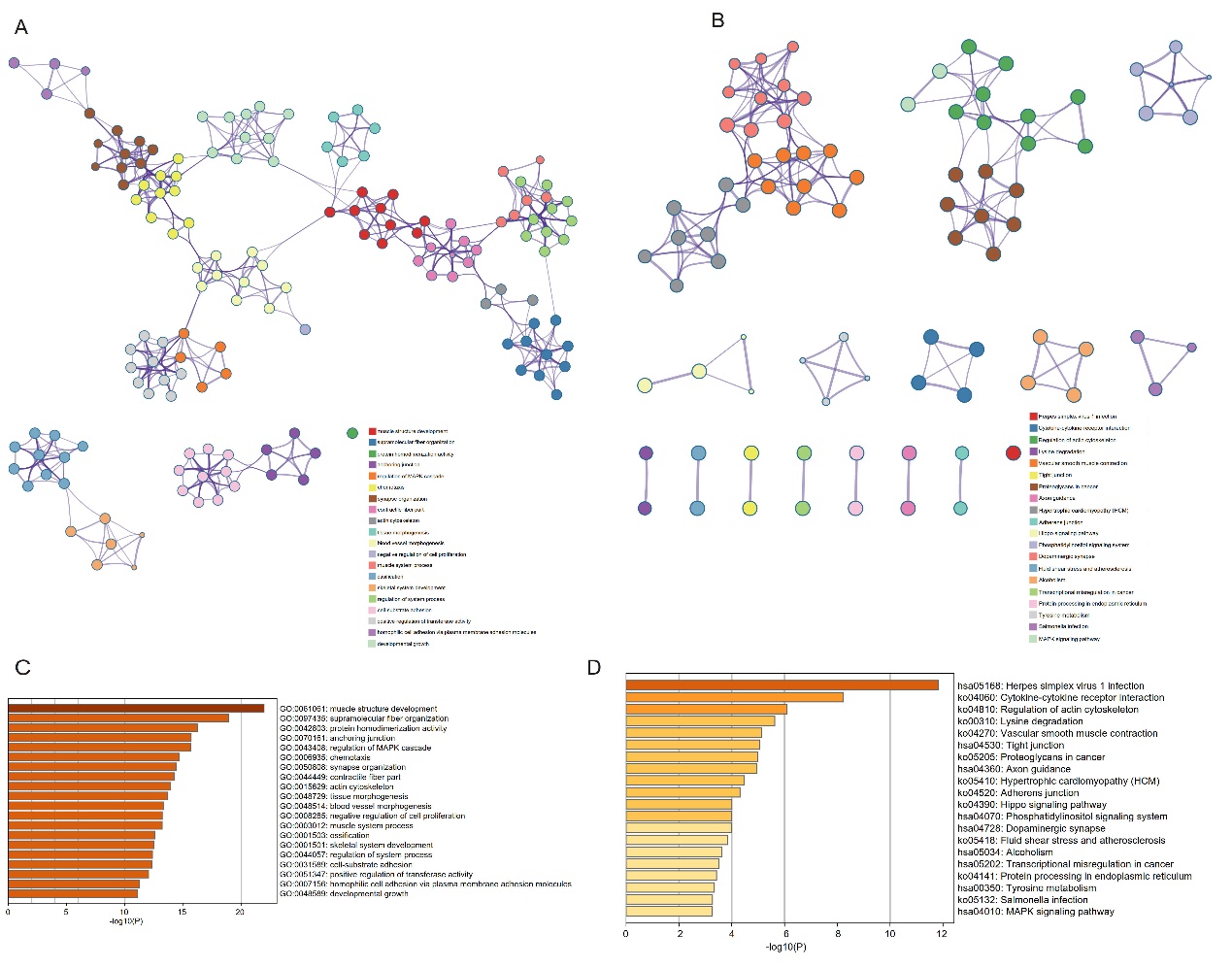


Figure S3. Gene functional enrichment analysis of the genes in blue module genes by Metascape. (A) Enrichment_GO _ColorByCluster analyses. (B) Enrichment_KEGG _ColorByCluster analyses. (C) Enrichment_heatmap_HeatmapSelected GO analyses. (D) Enrichment_heatmap_HeatmapSelected KEGG analyses.


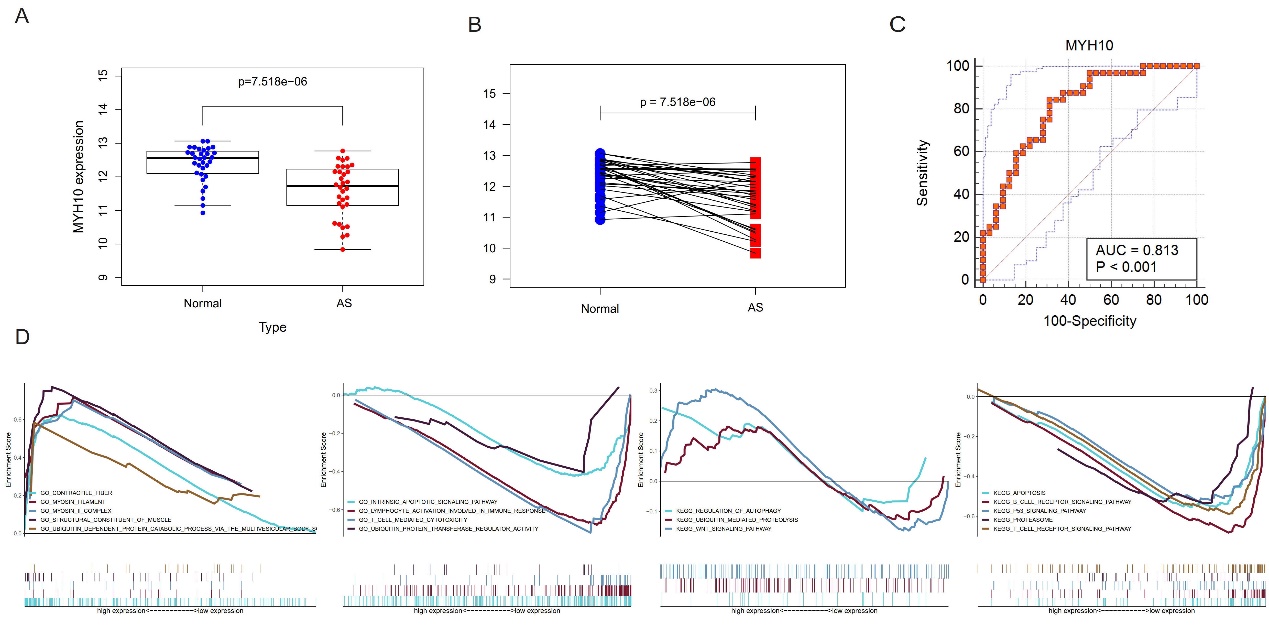


Figure S4. The single gene analysis of MYH10. (A) Performing differential expression analysis on MYH10 (B) Paired differential expression analysis on MYH10 (C) ROC curve shows the relationship between MYH10 and AS. (D) Gene functional enrichment analysis of MYH10 by GSEA.


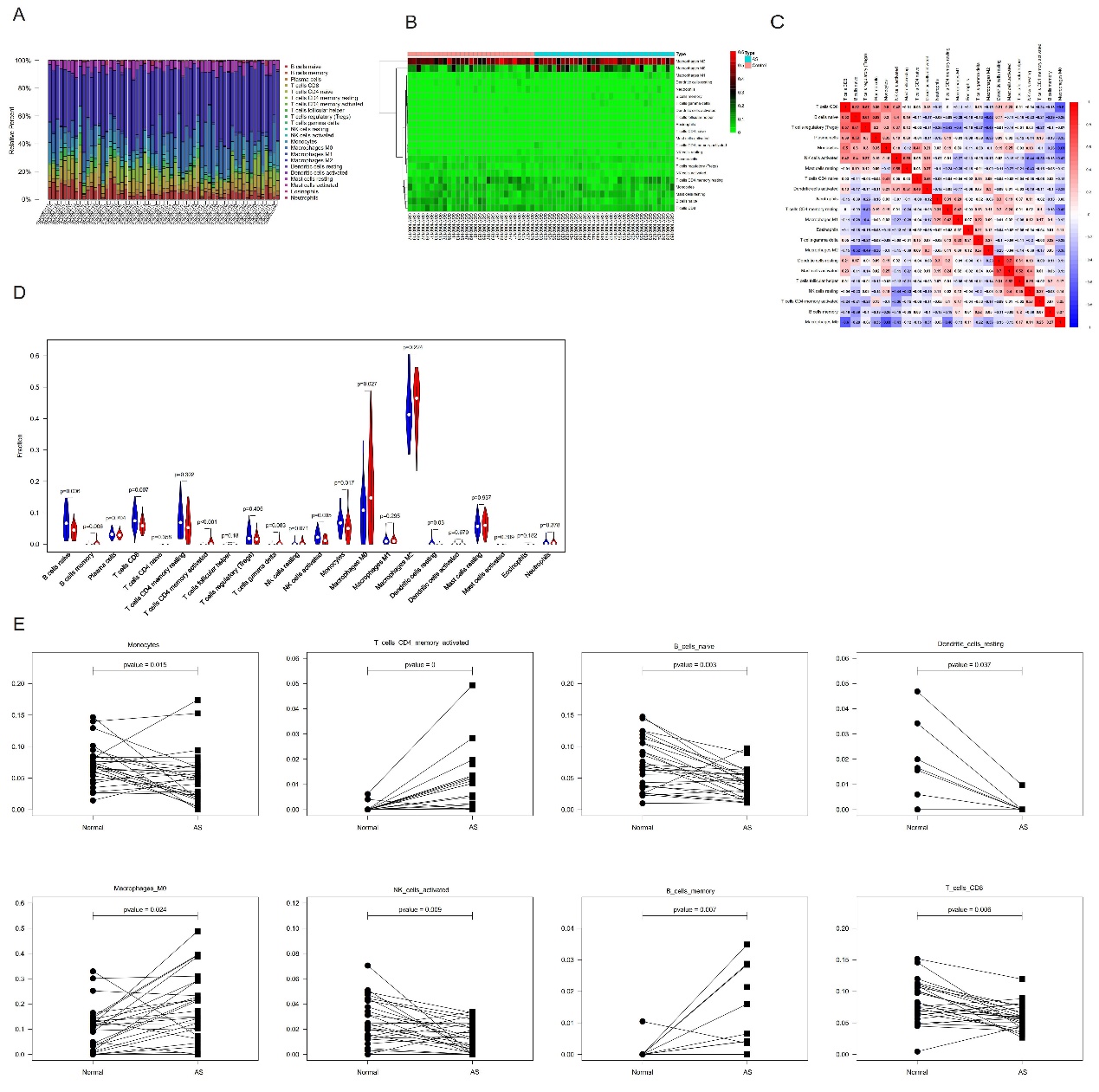


Figure S5. Composition of infiltrated immune cells between AS and normal tissues in the GEO cohort with CIBERSORT p < 0.05 for all eligible samples. 22 immune cells in GEO cohort were filtered for analyzing. (A) Fractions of immune cells in 32 normal and 32 AS samples in GEO. (B) Heatmap of 22 immune cells. (C) The correlational heatmap of 22 immune cells. (D) The violin plot of 22 immune cells between AS and normal tissues in GEO, the blue denotes normal tissue and the red denotes AS tissue. (E) Comparisons of 8 immune cells between AS and normal tissues. (P < 0.05)


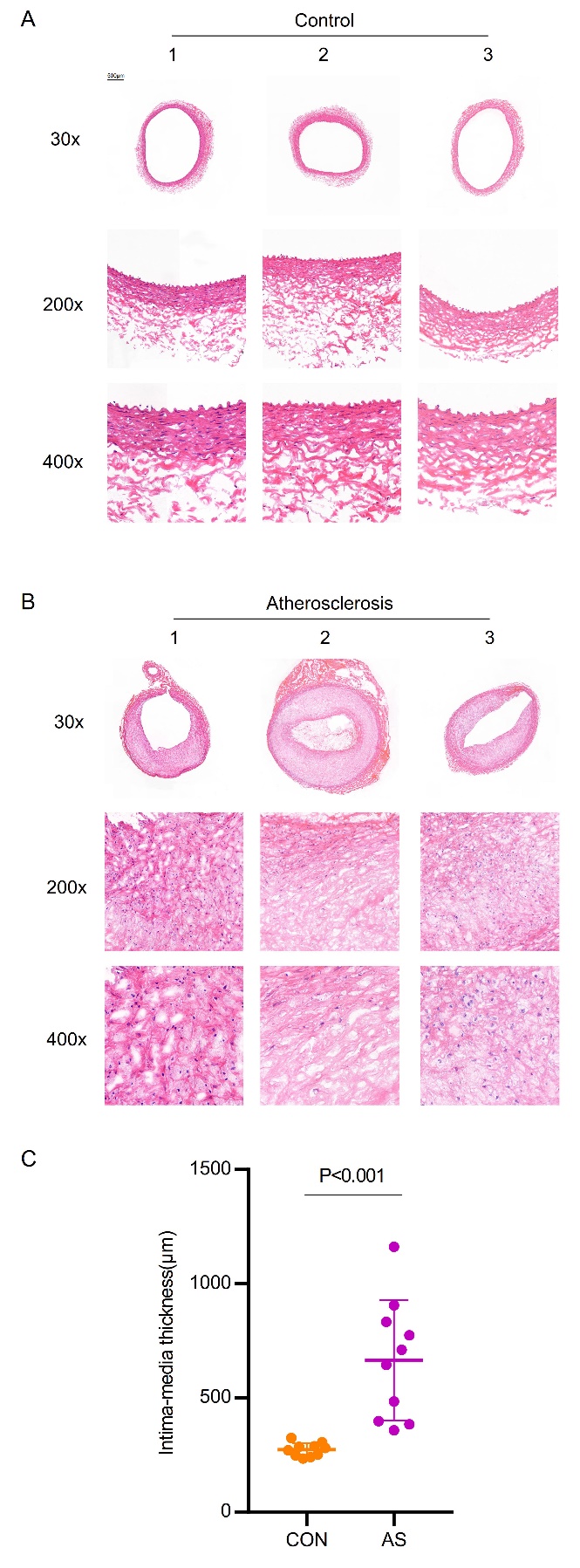


Figure S6. Successful construction of the AS-animal model. (A) The HE staining of the arterial sections in the control group (30x, 200x, 400x). (B) Arterial section in AS-animal model. (C) Quantitative analysis of intima-media thickness in the AS-animal model and control groups.


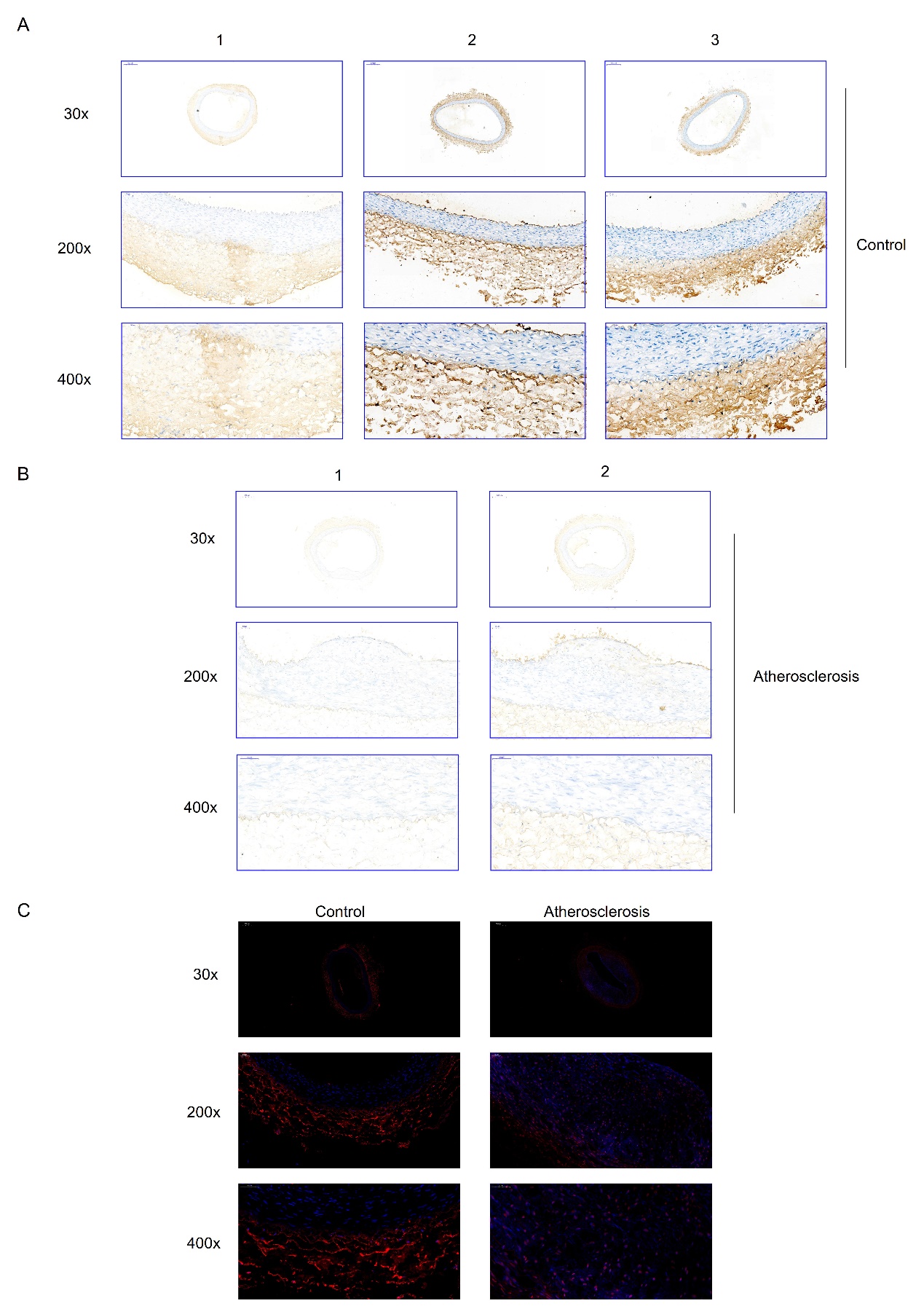


Figure S7. Expression of MYH10 via the immunohistochemical and immunofluorescence assay. (A) Immunohistochemical assay (30x, 200x, 400x) results showed that the MYH10 was clearly expressed in the media of the arteries. (B) In the AS-animal model, the MYH10 was down-expressed significantly in the media of arteries. (C) The immunofluorescence assay manifested the expression of MYH10.


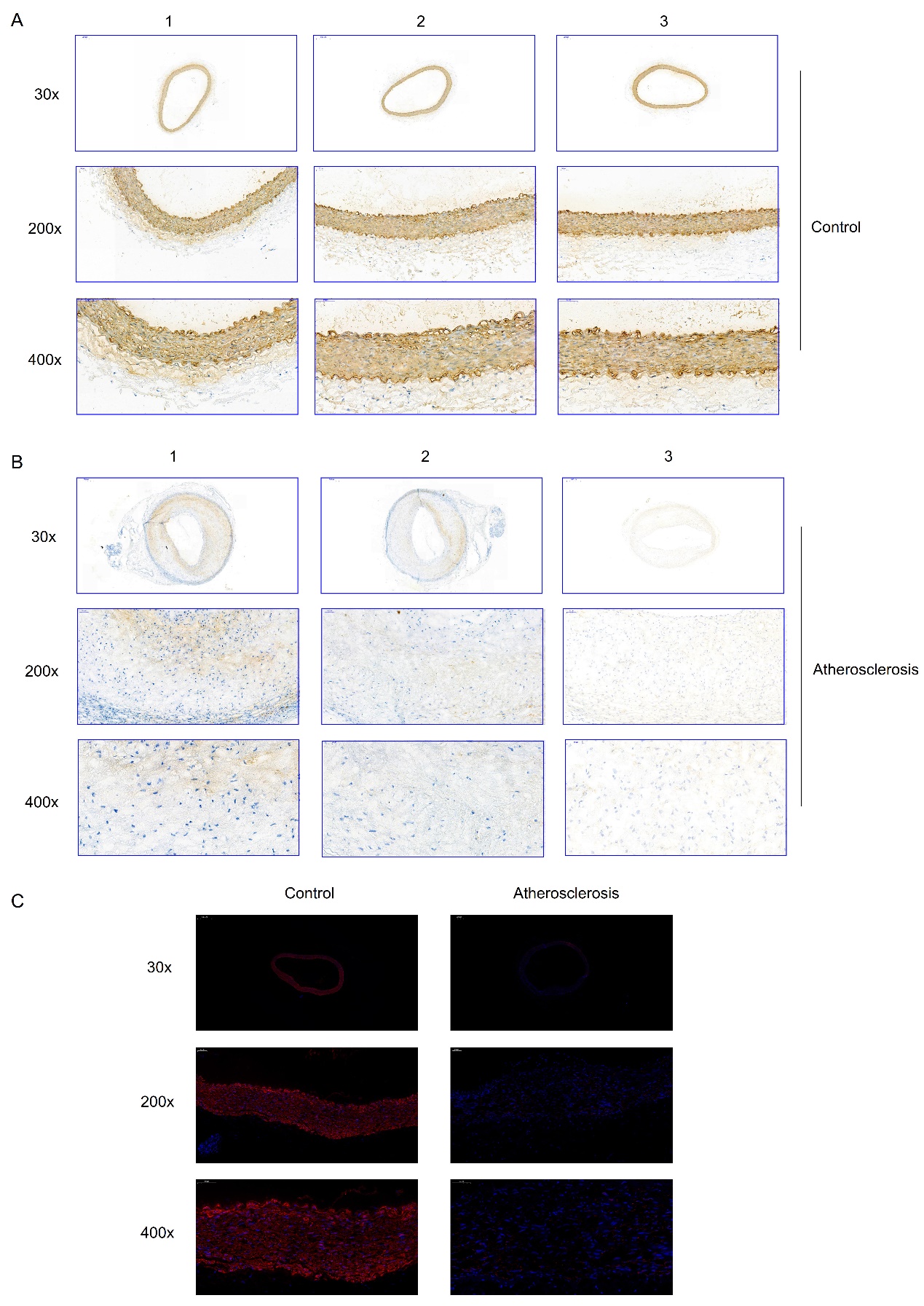


Figure S8. Expression of α-SMA via the immunohistochemical and immunofluorescence assay. (A) Immunohistochemical assay (30x, 200x, 400x) results showed that the α-SMA was clearly expressed in the media of the arteries. (B) In the AS-animal model, the α-SMA was down-expressed significantly in the media of arteries. (C) The immunofluorescence assay manifested the expression of α-SMA.


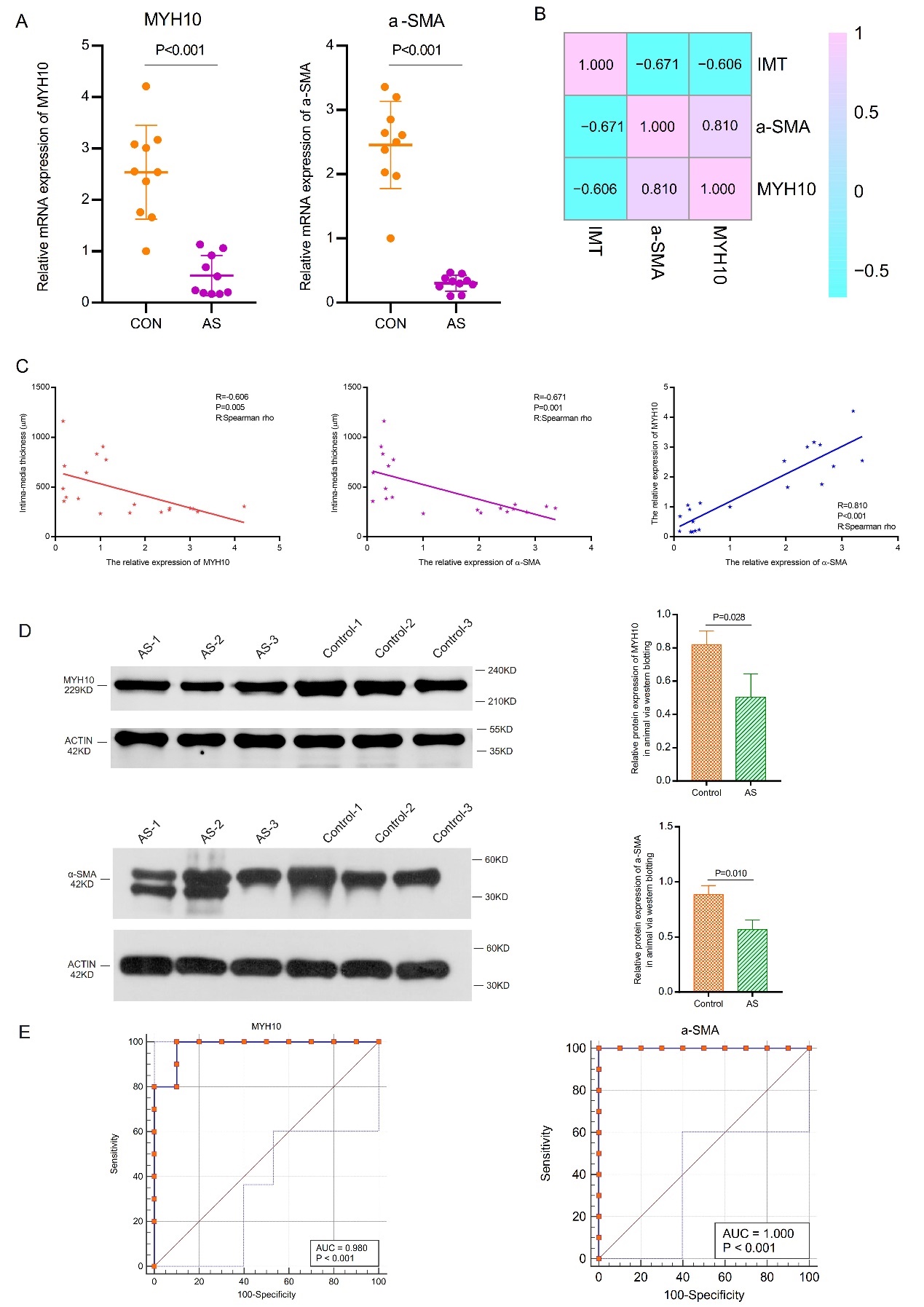


Figure S9. The expression of MYH10 and α-SMA via RT-PCR and western blotting, and strong associations among MYH10, α-SMA, and IMT in the animal model. (A) Compared with the control group, the expressions of MYH10 and α-SMA in the mRNA level were down-expressed in the AS-animal group significantly. (B) The heatmap showing the associations among MYH10, α-SMA, and IMT. (C) Strong associations among MYH10, α-SMA, and IMT via the scatter diagram. (D) In the Western blotting assay, the expressions of MYH10 and α-SMA were lower in the AS than the control group. (E) The results of ROC demonstrated that MYH10 or α-SMA expression could specifically and sensitively predict IMT.


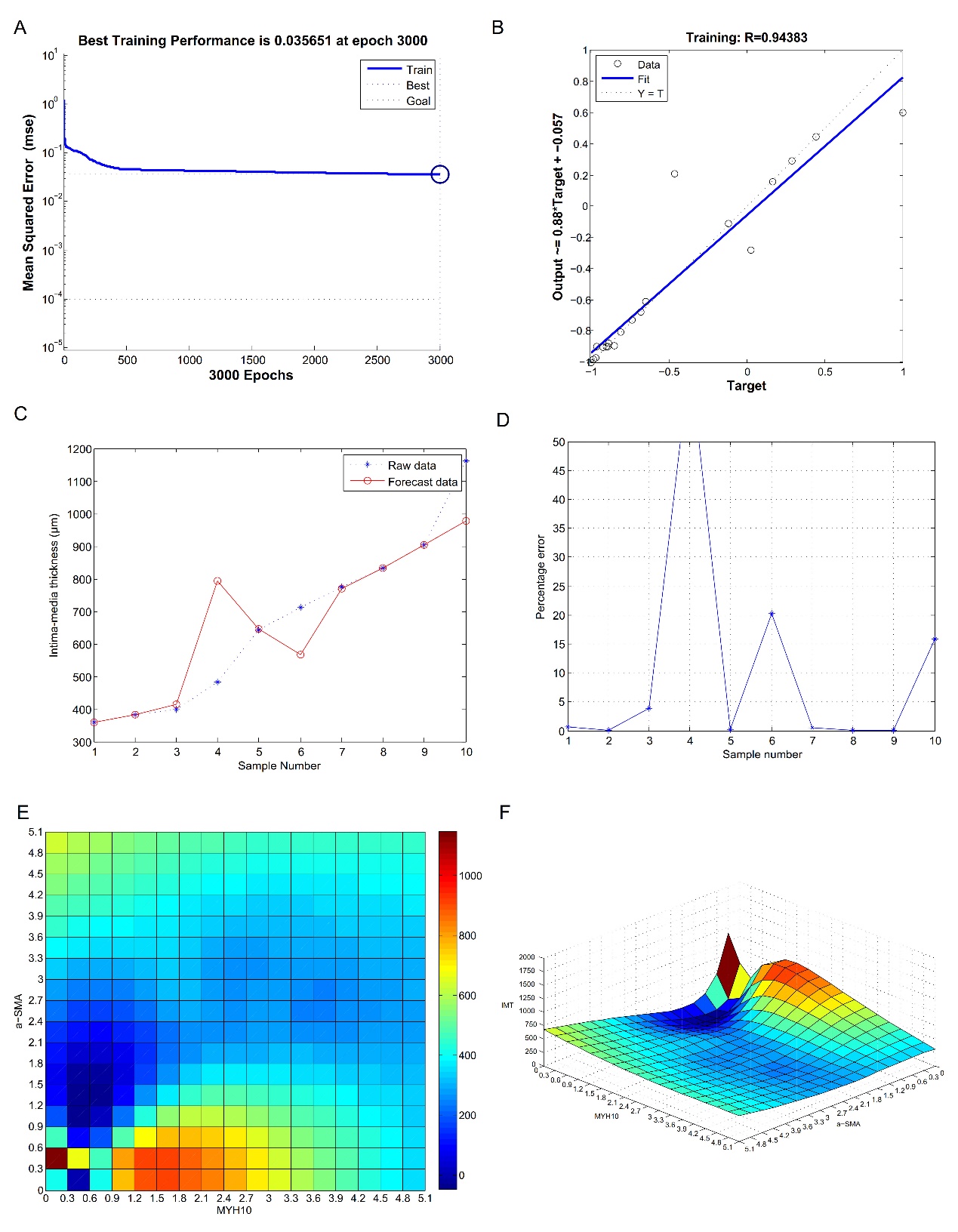


Figure S10. Prediction of IMT based on the neural network model and cubic spline interpolation algorithm in the animal model. (A) Best training performance is 0.035651 at epoch 3000. (B) The relativity of the model (built by MYH10, α-SMA and IMT) is 0.94383. (C, D) There exists tiny error between raw data and forecast data. (E, F) Results of cubic spline interpolation algorithm showed that the high-risk warning indicator of IMT of the AS: 0.90 < MYH10 < 2.70, and 0 < α-SMA < 0.90.


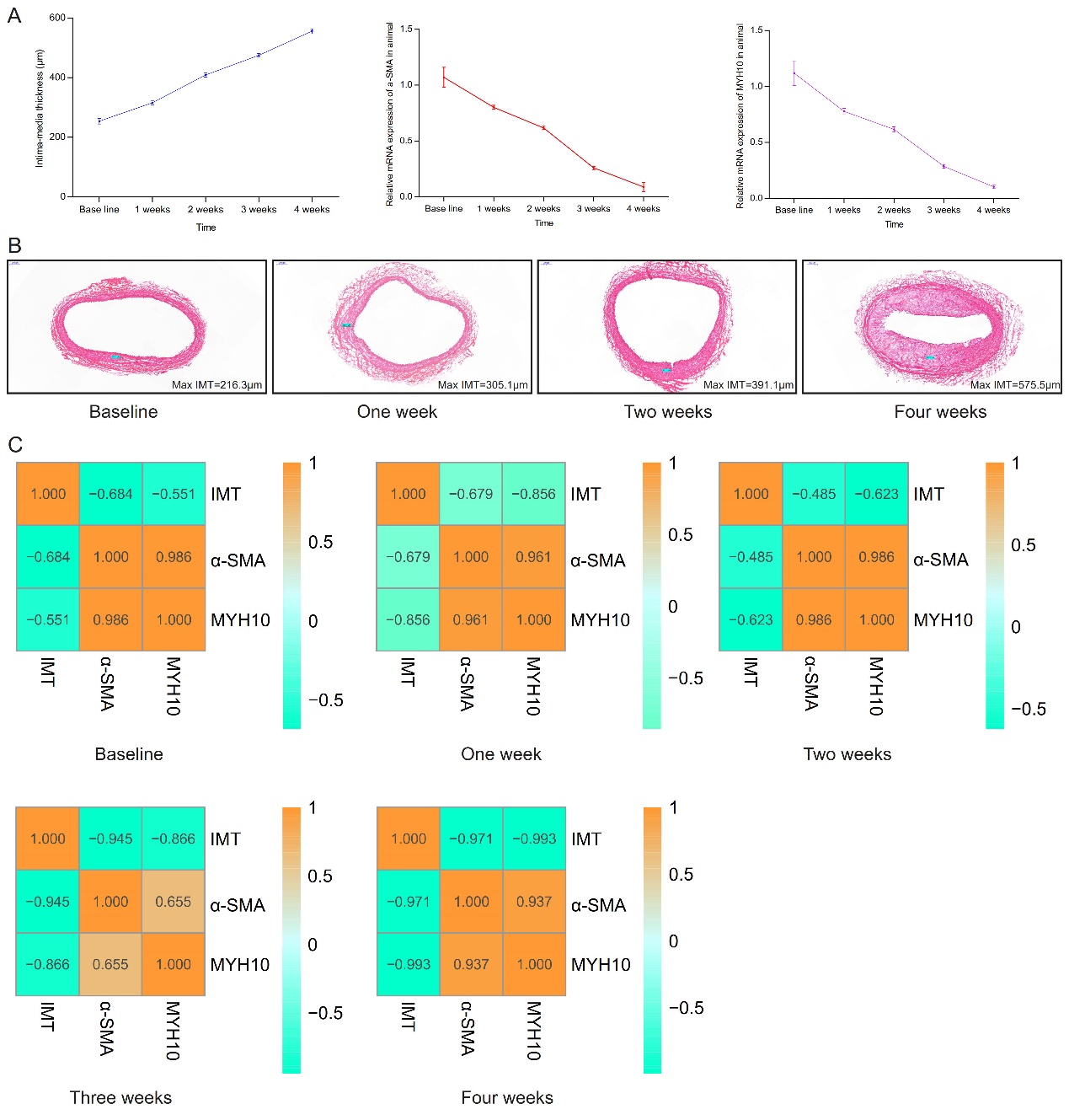


Figure S11. Gradual changes of intima-media thickness (IMT) in pathology, and the expression of α-SMA, MYH10, and the relationships among them at the different time points. (A) With the passage of time, the IMT gradually increased. However, with the passage of time, the expression of α-SMA and MYH10 gradually decreased. (B) Gradual changes of IMT in pathology. (C) The strong relationships among IMT, expression of α-SMA and MYH10 at the different time points. And there existed negative correlations between IMT and expression of α-SMA (and MYH10). However, the expression of MYH10 was positively related with the α-SMA.


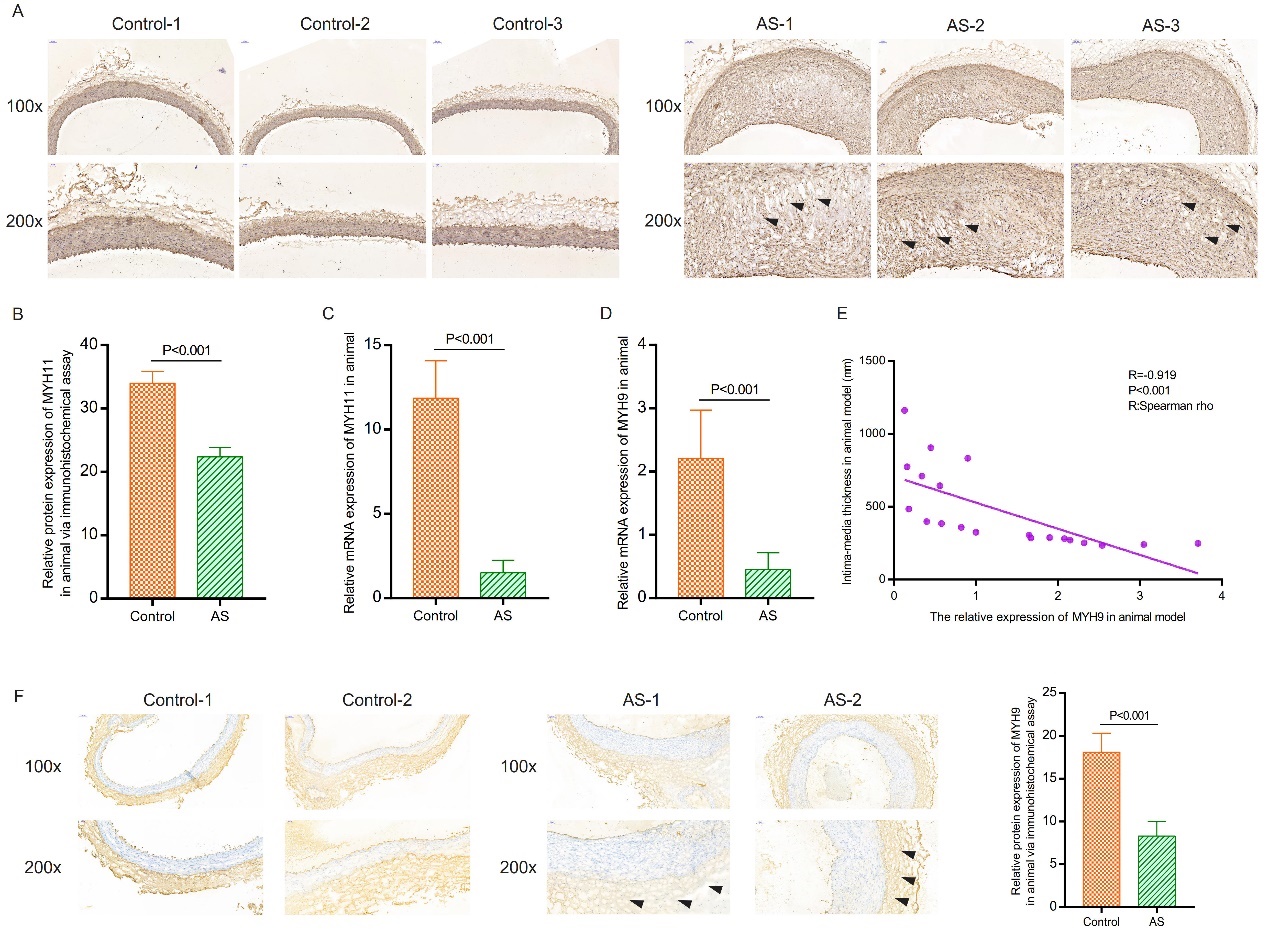


Figure S12. The expression of MYH11 and MYH9 in the control artery and atherosclerosis samples in the animal model via the immunohistochemical assay and RT-qPCR. (A, B) Through the immunohistochemical assay, the expression of MYH11 in control artery samples was up-regulated compared with the atherosclerosis samples (P<0.001). (C) And the RT-qPCR also verified that the expression of MYH11 in the atherosclerosis samples was lower than the control artery samples (P<0.001). (D) Through the RT-qPCR assay, the expression of MYH9 in control artery samples was up-regulated compared with the atherosclerosis samples (P<0.001). (E) The relative expression of MYH9 in the artery in animal model was negatively related with the intima-media thickness (R=-0.919, P<0.001). (F) Through the immunohistochemical assay, the expression of MYH9 in atherosclerosis samples was lower than the control artery samples (P<0.001).


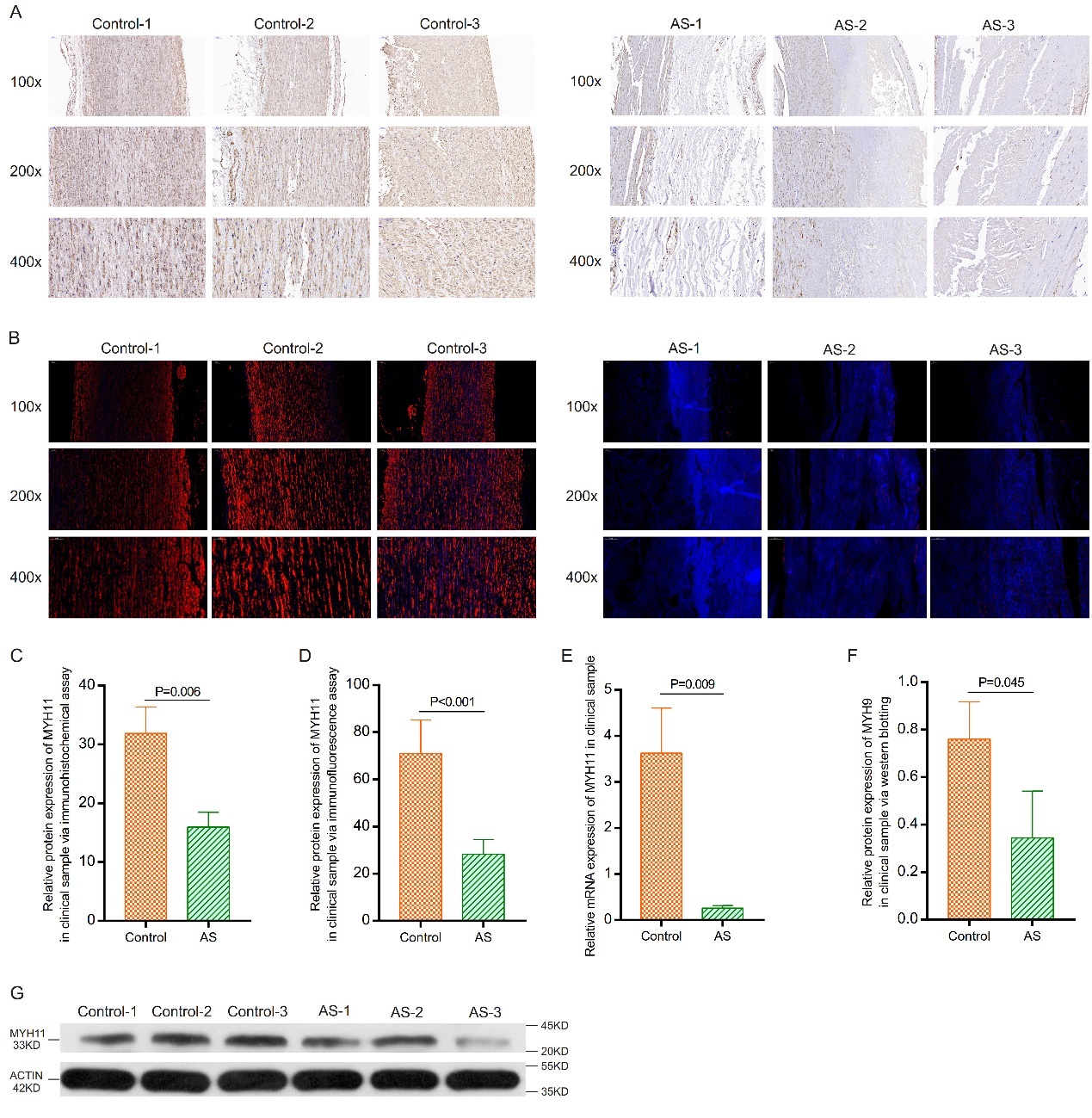


Figure S13. Verification for the role of MYH11 based on the clinical AS samples. (A) The immunohistochemical assay (100x, 200x, 400x) showed that the MYH11 was down-expressed significantly in the media of arteries in the clinical AS samples compared with the control group (The brown-yellow color represents the expression of the MYH11 molecule, and the blue color represents the nucleus). (B) The immunofluorescence assay showed that the MYH11 was down-expressed significantly in the media of arteries in the clinical AS samples compared with the normal group (The red color represents the expression of the MYH11 molecule, and the blue color represents the nucleus). (C) Quantitative analysis of relative protein expression of MYH11 in clinical sample via immunohistochemical assay between the control and AS samples (P=0.006). (D) Quantitative analysis of relative protein expression of MYH9 in clinical sample via immunofluorescence assay between the control and AS samples (P<0.001). (E) The RT-qPCR results indicated the relative expression level of MYH11 mRNA. (F, G) Western blotting analysis showed that the expression of MYH11 proteins was down-regulated in the clinical AS compared with the control group (P=0.045).


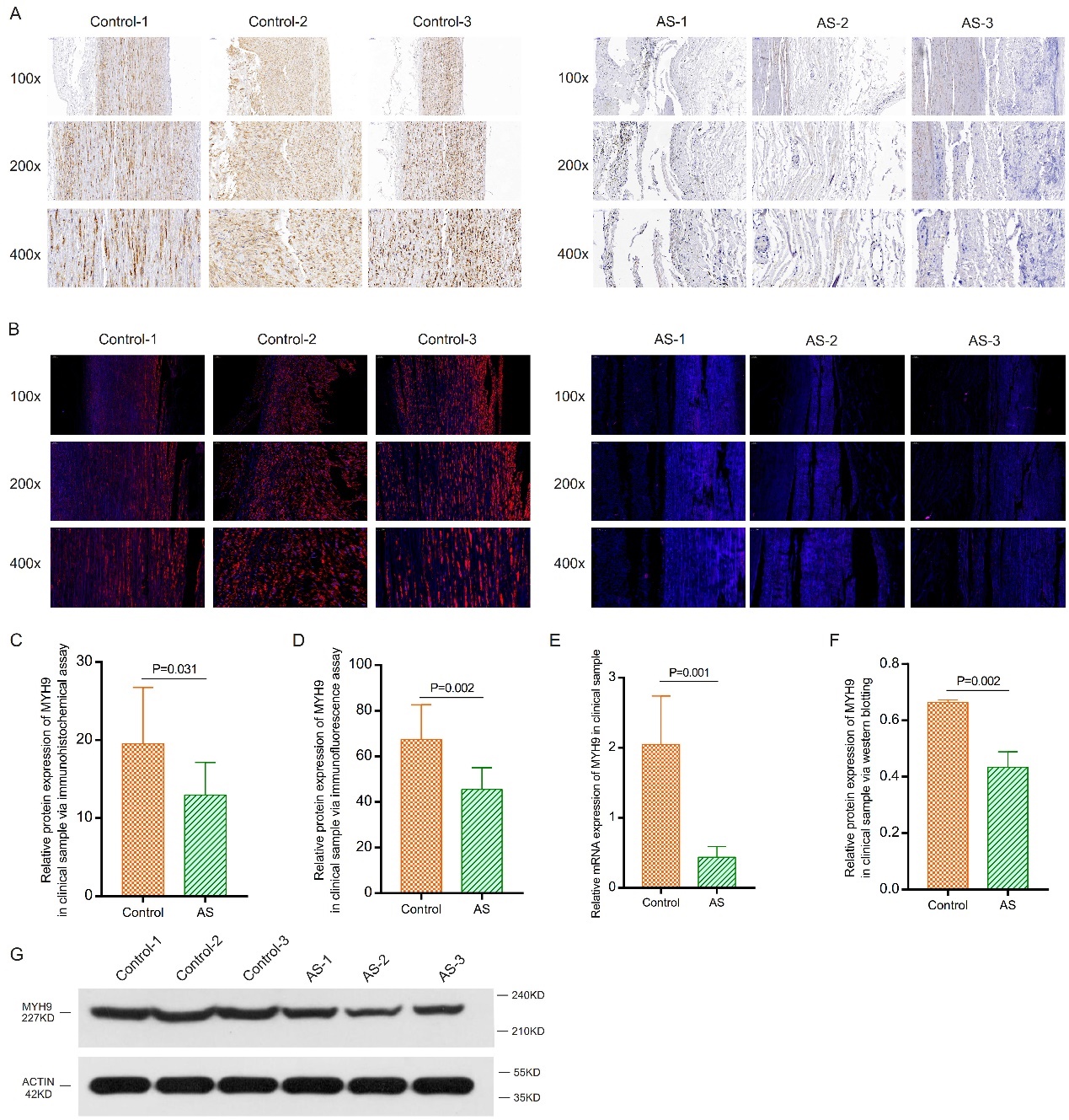


Figure S14. Verification for the role of MYH9 based on the clinical AS samples. (A) The immunohistochemical assay (100x, 200x, 400x) showed that the MYH9 was down-expressed significantly in the media of arteries in the clinical AS samples compared with the control group (The brown-yellow color represents the expression of the MYH9 molecule, and the blue color represents the nucleus). (B) The immunofluorescence assay showed that the MYH9 was down-expressed significantly in the media of arteries in the clinical AS samples compared with the normal group (The red color represents the expression of the MYH9 molecule, and the blue color represents the nucleus). (C) Quantitative analysis of relative protein expression of MYH9 in clinical sample via immunohistochemical assay between the control and AS samples (P=0.031). (D) Quantitative analysis of relative protein expression of MYH9 in clinical sample via immunofluorescence assay between the control and AS samples (P=0.002). (E) The RT-qPCR results indicated the relative expression level of MYH9 mRNA. (F, G) Western blotting analysis showed that the expression of MYH11 proteins was down-regulated in the clinical AS compared with the control group (P=0.002).


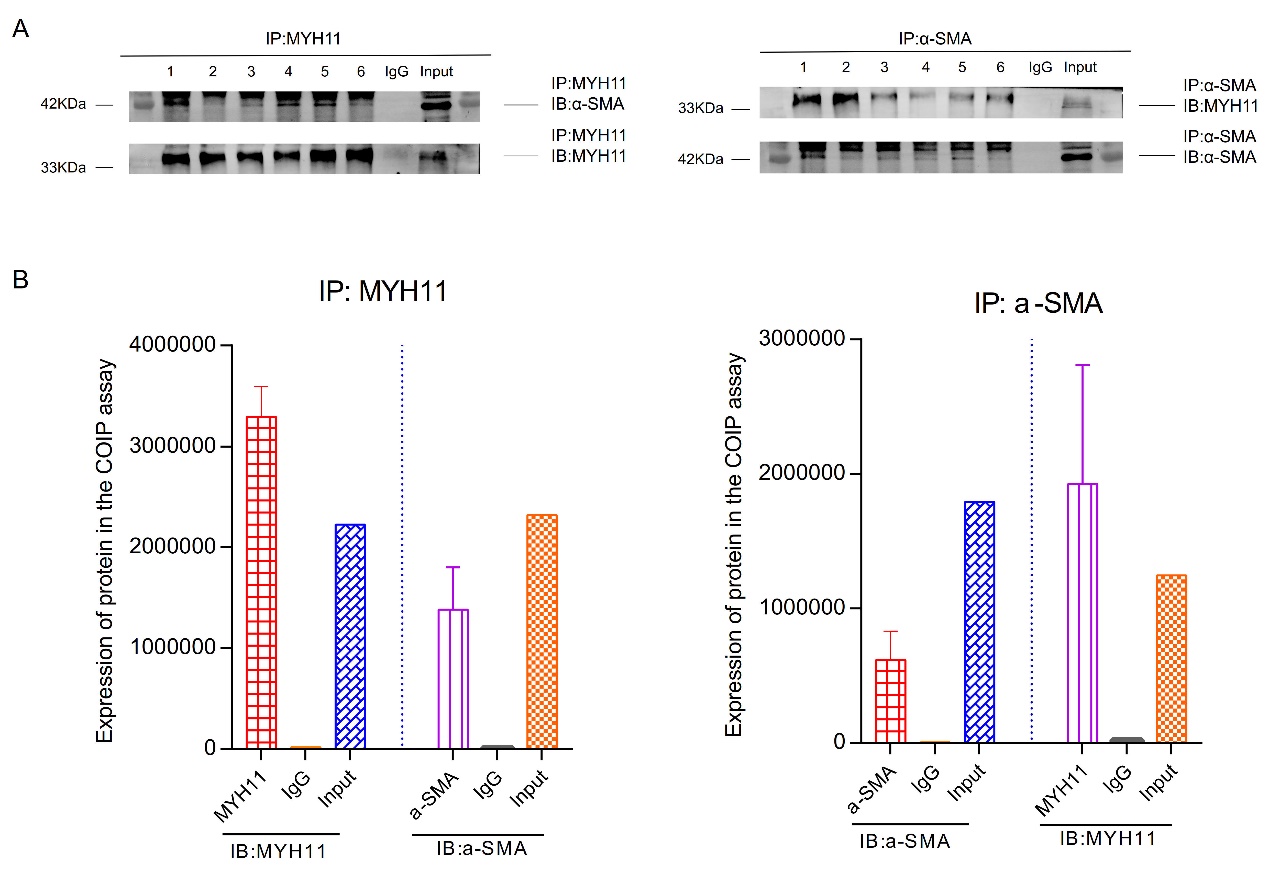


Figure S15. The Co-IP experiment manifested that the MYH11 and α-SMA were co-expressed in the artery.
